# The global succinylation of SARS-CoV-2–infected host cells reveals drug targets

**DOI:** 10.1073/pnas.2123065119

**Published:** 2022-07-12

**Authors:** Quan Liu, Heming Wang, He Zhang, Liyan Sui, Letian Li, Wang Xu, Shouwen Du, Pengfei Hao, Yuhang Jiang, Jing Chen, Xiaoyun Qu, Mingyao Tian, Yinghua Zhao, Xuerui Guo, Xingye Wang, Wu Song, Guangqi Song, Zhengkai Wei, Zhijun Hou, Guoqing Wang, Minhua Sun, Xiao Li, Huijun Lu, Xinyu Zhuang, Ningyi Jin, Yicheng Zhao, Chang Li, Ming Liao

**Affiliations:** ^a^Key Laboratory of Livestock Disease Prevention of Guangdong Province, Scientific Observation and Experiment Station of Veterinary Drugs and Diagnostic Techniques of Guangdong Province, Ministry of Agriculture and Rural Affairs, Institute of Animal Health, Guangdong Academy of Agricultural Sciences, 510610 Guangzhou, China;; ^b^Center for Infectious Diseases and Pathogen Biology, Key Laboratory of Organ Regeneration and Transplantation of the Ministry of Education, State Key Laboratory of Human-Animal Zoonotic infectious Diseases, The First Hospital of Jilin University, Changchun, 130021 China;; ^c^Research Unit of Key Technologies for Prevention and Control of Virus Zoonoses, Chinese Academy of Medical Sciences, Changchun Veterinary Research Institute, Chinese Academy of Agricultural Sciences, Changchun, 130122 China;; ^d^School of Life Sciences and Engineering, Foshan University, 528011 Foshan, China;; ^e^Department of Gastroenterology, Zhongshan Hospital, Fudan University, 200437 Shanghai, China;; ^f^Department of Infectious Diseases, The Second Clinical Medical College (Shenzhen People’s Hospital) of Jinan University, 518020 Shenzhen, China;; ^g^Key Laboratory of Zoonosis of Ministry of Agriculture and Rural Affairs, South China Agricultural University, 510642 Guangzhou, China;; ^h^School of Pharmacy, Jilin University, 130012 Changchun, China;; ^i^Clinical Medical College, Changchun University of Chinese Medicine, 130117 Changchun, China;; ^j^Shanghai Institute of Liver Diseases, 200032 Shanghai, China;; ^k^College of Wildlife and Protected Area, Northeast Forestry University, 150040 Harbin, China;; ^l^Department of Pathogenbiology, College of Basic Medicine, Jilin University, 130012 Changchun, China;; ^m^Maoming Branch, Guangdong Laboratory for Lingnan Modern Agriculture, 525000 Maoming, China

**Keywords:** SARS-CoV-2, succinylproteomics, antiviral, SIRT5, NSP14

## Abstract

SARS-CoV-2, the causative agent of the COVID-19 pandemic, undergoes continuous evolution, highlighting an urgent need for development of novel antiviral therapies. Here we show a quantitative mass spectrometry-based succinylproteomics analysis of SARS-CoV-2 infection in Caco-2 cells, revealing dramatic reshape of succinylation on host and viral proteins. SARS-CoV-2 infection promotes succinylation of several key enzymes in the TCA, leading to inhibition of cellular metabolic pathways. We demonstrated that host protein succinylation is regulated by viral nonstructural protein (NSP14) through interaction with sirtuin 5 (SIRT5); overexpressed SIRT5 can effectively inhibit virus replication. We found succinylation inhibitors possess significant antiviral effects. We also found that SARS-CoV-2 nucleocapsid and membrane proteins underwent succinylation modification, which was conserved in SARS-CoV-2 and its variants. Collectively, our results uncover a regulatory mechanism of host protein posttranslational modification and cellular pathways mediated by SARS-CoV-2, which may become antiviral drug targets against COVID-19.

## Significance

The continued rapid evolution of SARS-CoV-2 and the emergence of immune-evading variants pose significant challenges to COVID-19 prevention and control, highlighting the urgent need for development of novel antiviral therapies. Our study found that SARS-CoV-2 infection promotes host succinylation and inhibits several key enzymes of the TCA, a crucial metabolic pathway that connects carbohydrate, fat, and protein metabolism, as well as regulating cellular energy. Additionally, viral NSP14 is capable to participate in succinylation through interacting with host SIRT5, a cellular desuccinylase. It is noteworthy that succinylation inhibitors can significantly reduce the viral replication, as a potential guide for the treatment of COVID-19.

Since its emergence in 2019, SARS-CoV-2 has caused an ongoing pandemic of COVID-19 worldwide (1). SARS-CoV-2, together with the bat-origin coronavirus SARS-CoV and Middle East respiratory syndrome coronavirus (MERS-CoV), usually induce respiratory diseases (2). By comparison, SARS-CoV-2 has high transmissibility, and has continued to evolve, leading to the emergence of vaccine-breakthrough variants (3–5). Effective and safe antiviral drugs are limited in the treatment of COVID-19. Thus, it is necessary to develop effective and broad-spectrum antiviral drugs for SARS-CoV-2 and emerging variants (6–8).

Protein posttranslational modifications (PTMs) are the critical regulation mechanisms of many cellular processes, which makes them attractive viral targets. As viruses cannot replicate themselves, they have developed abilities to alter cellular pathways and proteins required for virus replication, including protein PTMs. Lysine residues in proteins are vulnerable to a variety of PTMs—such as methylation, acetylation, ubiquitination, and succinylation—which play important roles in cellular physiology and pathology. Succinylation, a newly identified PTM, endows lysine group with two negative charges, which leads to more changes in protein structure and function (9). Quantitative proteomics has been widely used to clarify viral pathogenesis by explaining the changed cellular processes and identifying the pivotal pathways or proteins that are potential antiviral targets (10). Here we present the global succinylation and protein abundance in SARS-CoV-2–infected cells and map these succinylation changes to the disturbed pathways. We also determine the possible mechanism of host succinylation regulated by SARS-CoV-2 and evaluate potential drugs to treat COVID-19.

## Results

### Succinylation as an Important PTM in SARS-CoV-2–Infected Cells.

PTMs are considered as emerging critical regulators of biological mechanisms and potential antiviral drug targets in infectious diseases (11–13). To explore the effect of SARS-CoV-2 infection on host PTMs during the early infection, we first determined the viral infection conditions (multiplicity of infection [MOI] of 0.01 for 0, 12, and 24 h) in Caco-2 cells, a cell line that originated from the colon of a colorectal adenocarcinoma patient, which is permissive to SARS-CoV-2 infection (*SI Appendix*, Fig. S1) (14). We examined the overall profiles of five PTM types upon SARS-CoV-2 infection, including ubiquitination, acetylation, malonylation, succinylation, and lactoylation, respectively. Interestingly, we found that the protein succinylation was significantly up-regulated during the early infection, which was positively correlated with the period of viral infection (*SI Appendix*, Fig. S2).

**Fig. 1.**
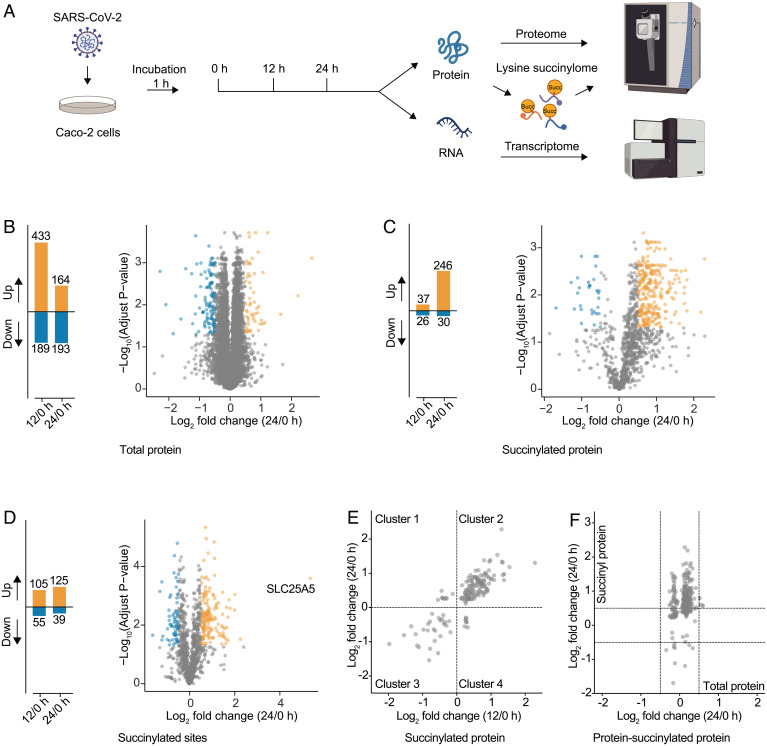
Global proteomics of succinylation and abundance upon SARS-CoV-2 infection. (*A*) Workflow of multiomics of SARS-CoV-2–infected cells. Infected Caco-2 cells were harvested at 0, 12, and 24 hpi, and the lysates were subjected to transcriptomics, quantitative proteomics, and succinyl-proteomics analysis. (*B*) Protein abundance in cells upon viral infection. The increased (orange) or decreased (blue) protein number across the infection course (*Left*). Volcano plot of protein abundance quantification at 24 hpi as comparison with 0 hpi (*Right*). (*C*) Succinylated protein abundance in cells upon viral infection. The increased (orange) or decreased (blue) number of succinylated proteins across the infection course (*Left*). Volcano plot of succinylated protein abundance quantification at 24 hpi as comparison with 0 hpi (*Right*). (*D*) Succinylated sites in cells upon viral infection. The increased (orange) or decreased (blue) number of succinylated sites across the infection course (*Left*). Volcano plot of succinylated sites at 24 hpi as comparison with 0 hpi (*Right*). We defined log_2_ fold-change > 0.5 as up-regulated proteins/sites (*P* < 0.01), and log_2_ fold-change < −0.5 as down-regulated proteins/sites (*P* < 0.01). SLC25A5, the mitochondrial solute carrier family 25, was the most up-regulated protein. (*E*) Changed succinylated proteins across the infection course. Scatterplot showed the fold-change of succinylated proteins in infected cells at 12/0 h (*x* axis) and 24/0 h (*y* axis). The dashed line represents log_2_ fold-change of 0. (*F*) Merged succinylated protein abundance with total protein abundance in infected cells. Scatterplot showed the fold-changes of total protein abundance in infected cells at 24/0 h (*x* axis) and succinylated protein abundance at 24/0 h (*y* axis). The dashed line represents log_2_ fold-change of ± 0.5.

We further focused on succinylome of SARS-CoV-2–infected host cells. Caco-2 cells were harvested in biological triplicate at 0, 12, and 24 h postinfection (hpi). Each sample was analyzed for detecting the changes in global protein abundance, succinylation, or transcription (Fig. 1*A* and *SI Appendix*, Fig. S3 and Datasets S1–S3). The infection group at 0 hpi was used for all comparisons to calculate the fold-changes of proteins or genes.

As expected, host proteins were decreased in abundance over the infection time course (Fig. 1*B*), which is a common phenomenon of viral infections, as viruses can inhibit nuclear export and translation of host mRNA (15, 16). The abundance of succinylated proteins and sites was consistently increased, implying that host succinylated proteins responded to the viral infection (Fig. 1 *C* and *D* and *SI Appendix*, Fig. S4). We divided these host succinylated proteins into four clusters according to the fold-changes in abundance, and found that many succinylated proteins were located in cluster 2, showing that succinylation levels of host proteins were positively correlated to the process of viral infection, which were further confirmed by deeper analysis in nine clusters of protein abundance and succinyl protein abundance during the infection (12/0 h compared to 24/0 h) (Fig. 1*E*, *SI Appendix*, Figs. S5 and S6, and Datasets S4 and S5). In order to eliminate the interference of nonsuccinylated proteins, we compared the fold-changes of total proteins and succinylated proteins in abundance. As shown in Fig. 1*F*, the abundance of the host succinylated proteins were significantly up-regulated at 24 hpi, while the abundance of total proteins did not change remarkably. These findings suggested that protein succinylation may represent an important host response during the SARS-CoV-2 infection.

### Succinylation of SARS-CoV-2 Protein.

To detect the translation pattern of SARS-CoV-2 protein, we analyzed the viral transcriptome and proteome abundance at different time points. Most viral transcripts were significantly up-regulated at the early stage of infection (*SI Appendix*, Fig. S7 *A*–*C*). Regarding the viral protein abundance, only four proteins, including nucleocapsid (N), membrane glycoprotein (M), spike protein (S), and open reading frame 3 (ORF3), were detectable at 12 hpi, while the other viral proteins were detectable at 24 hpi (*SI Appendix*, Figs. S7*D* and S8). These results were consistent with the life cycle of SARS-CoV-2 in infected cells. After rapid replication of the viral genome at the early stage, host translation elements are utilized to initiate the synthesis of viral proteins (17).

**Fig. 2.**
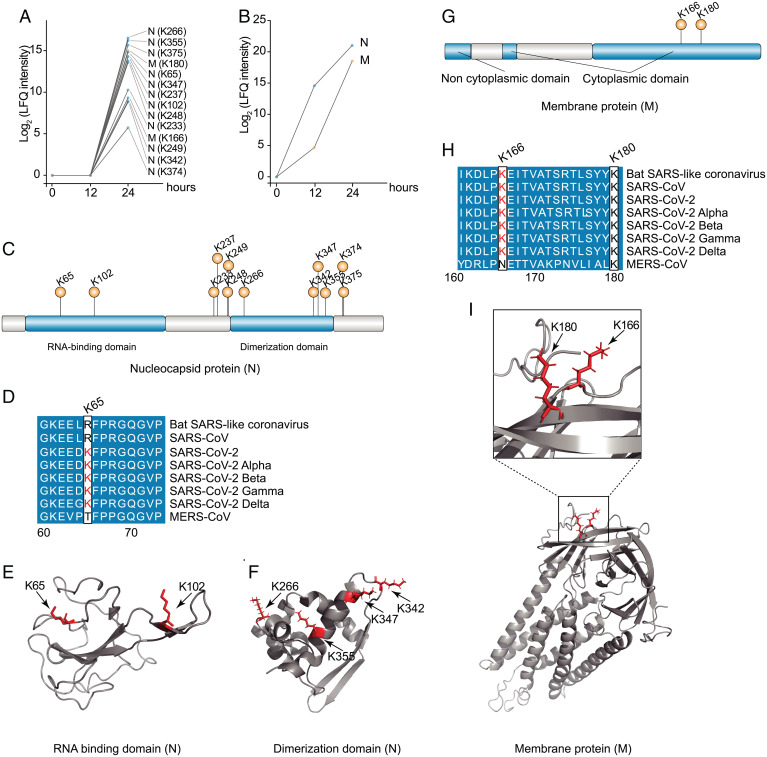
Succinylation of SARS-CoV-2 N and M proteins. (*A*) Succinylated sites in the SARS-CoV-2 N and M proteins. The number represents the location of succinylation. M contained two succinylated sites, and N contained 12 succinylated sites. The viral protein abundance was expressed as label-free quantification (LFQ). (*B*) Abundance of M and N proteins in SARS-CoV-2-infected cells during the infection course. (*C*) Succinylated sites in the N protein of SARS-CoV-2. Two structural domains of RNA binding domain and dimerization domain predicted by InterPro are shown (51). (*D*) Amino acid sequence alignment of N protein succinylated site K65 between SARS-CoV-2 and other coronaviruses. Other succinylated sites of N protein are shown in *SI Appendix*, Fig. S9. (*E* and *F*) The succinylated site was mapped to the crystal structure of N protein RNA binding domain (*E*) and dimerization domain (*F*). (*G*) Succinylated sites K166 and K180 in the M protein of SARS-CoV-2. (*H*) Amino acid sequence alignment of M protein from SARS-CoV-2 and coronaviruses. (*I*) The succinylated sites were mapped to the crystal structure of M protein. Two succinyl lysine sites were highlighted within the crystal structure of SARS-CoV-2 M that was in silico predicted by AlphaFold (52).

PTMs of the viral proteins may affect virus replication, assembly, and release during the infection (18–20). We found that succinylation modification only occurred in viral M and N proteins at 24 hpi (Fig. 2*A*). In contrast, both M and N proteins displayed high abundance at 12 hpi, but succinylation modification was absent at this time (Fig. 2 *A* and *B*), indicating that succinylation of M and N proteins occurred after protein translation during the course of viral replication (12 to 24 hpi).

We found that M protein contained 2 succinylated sites and N had 12 succinylated sites (Fig. 2 *C* and *G*). The two succinylated sites (K65 and K102) were located in the RNA binding domain on the N protein, and the other 10 succinylated sites were located in or nearby the dimerization domain of N protein, which may affect the dimerization of N protein (21). All succinylated sites of N protein were highly conserved (*SI Appendix*, Fig. S8), with the exception of K65, which was unique in SARS-CoV-2 and its variants, instead of arginine in SARS-CoV, SARS-related coronaviruses, and threonine in MERS-CoV (*SI Appendix*, Fig. S8). Since the amino acids lysine (K) and arginine (R) are positively charged polar, succinylation is capable of converting their original properties, and affects the function of N protein in the life cycle of SARS-CoV-2.

The two succinylated sites were located in the cytoplasmic domain of the M protein, and conserved in SARS-like coronaviruses (Fig. 2 *G* and *H*). Phosphorylation and ubiquitination have also been found in the succinylated sites on N and M proteins (12, 22). We further mapped these succinylated sites to the available domain structures of N protein and crystal structure of M protein, and found that they were all located on the surface of available crystal structures (Fig. 2 *E*, *F*, and *I*). These findings suggest that SARS-CoV-2 protein PTMs, including succinylation, may play a regulatory role during virus replication.

### Host Proteins Succinylation upon SARS-CoV-2 Infection.

We identified 1,000 succinylated host proteins (2,142 succinylated sites) in cells at 24 hpi, and 246 proteins (1,015 succinylated sites) were differentially expressed. Enrichment analysis showed that the up-regulated succinylated host proteins were involved in ribosome, spliceosome, proteasome metabolism-associated pathways, and down-regulated succinylated proteins were related to systemic lupus erythematosus, alcoholism, neutrophil extracellular trap formation, and viral carcinogenesis (*SI Appendix*, Fig. S9 and Dataset S6).

**Fig. 3.**
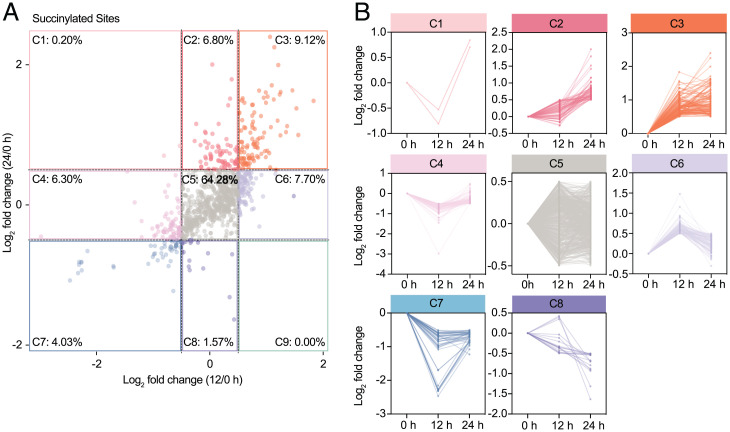
Cluster analysis of host protein succinylation sites upon SARS-CoV-2 infection. (*A*) Scatterplot illustrated all succinylated sites were divided into nine clusters based on the log_2_ fold-changes of ± 0.5 in 12/0h (*x* axis) compared to 24/0h (*y* axis). The dashed lines represent log_2_ fold-change of ± 0.5. (*B*) Changes of the succinylated sites in each cluster at 12 and 24 h compared with 0 h post infection. The color of each cluster in *B* corresponds to that in *A*.

We further divided the differentially expressed succinylation sites into nine clusters based on the log_2_ fold-change in 12/0 h compared with 24/0 h (Fig. 3 and Dataset S7), and found that the percentage of clusters 2, 3, and 6 (late, persistent, and early increased succinylation) could reach 23.6%, twice fold of the inhibition clusters 4, 7, 8, and 9 (12.1%), indicating that more and more succinylated sites occurred during the course of virus infection. This phenomenon is consistent with increased host protein succinylation (Fig. 1*E*). Moreover, several host proteins of cluster 3 were involved in virus–host interaction—such as CS, PMPCB, PMPCA, PPA2, ATP5PD, and HSPA9 (Fig. 3*B*)—which were detected by enrichment analysis, showing that they were mostly distributed in the mitochondria responsible for TCA cycle, fatty acid metabolism, ADP/ATP transport, and the electron transport chain (*SI Appendix*, Fig. S10).

We mapped host succinylation proteins to the interactome SARS-CoV-2 (13, 23, 24), and found the succinylation levels of 66 lysine residues on 25 host proteins were correlated with the viral infection. These host proteins have been reported to interact with structural proteins (E, M, and N) and nonstructural proteins (Nsp3, Nsp4, Nsp6, Nsp8, Orf3, Orf3a, Orf8, and Orf9c) of SARS-CoV-2, respectively (*SI Appendix*, Fig. S11), suggesting that virus proteins may disturb host succinylation by directly binding to host proteins.

### Succinylation of the Key Enzymes in Host Metabolism Pathways.

**Fig. 4.**
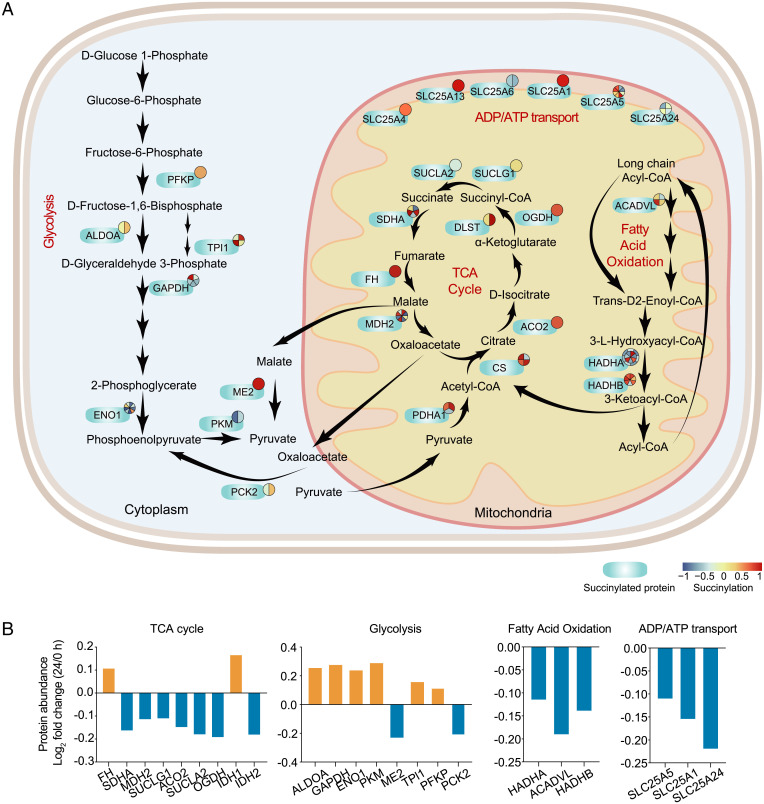
Succinylation of enzymes associated with metabolic pathways upon SARS-CoV-2 infection. (*A*) Succinylated enzymes involved in carbon metabolisms of glycolysis, TCA cycle, and fatty acid oxidation, and mitochondrial transport. The blue ovals represent succinylated proteins, the numbers of pie charts represent the succinylated sites, and the color of pie chart represents the succinylated sites level at 24 h compared to 0 h after infection. The enzymes for glycolysis included PFKP, ALDOA, TPI1, GAPDH, ENO1, ME2, PKM, and PCK2. The enzymes for the TCA cycle included CS, ACO2, DLST, SUCLG1, SDHA, FH, OGDH, MDH2, IDH1, IDH2, and PDHA1. The enzymes for fatty acid oxidation contained ACADVL, HADHA and HADHB. The enzymes for mitochondrial transport included SLC25A1, SLC25A4, SLC25A5, SLC25A6, SLC25A13, and SLC25A24. (*B*) Fold-changes of succinylated proteins in abundance at 24 h postinfection as comparison with 0 h postinfection. Nine succinylated proteins in TCA cycle, eight succinylated proteins in glycolysis, three succinylated proteins in ADP/ATP transport, and three succinylated proteins in fatty acid oxidation are shown.

We further analyzed the changes in succinylated levels of the key enzymes responsible for the TCA cycle, glycolysis, fatty acid oxidation, and mitochondrial (ADP/ATP) transport (Fig. 4). There were nine succinylated proteins involved in the TCA cycle—including CS, ACO2, DLST, SUCLG1, SUCLA2，SDHA, FH, OGDH, and MDH2—which had hypersuccinylated sites (Fig. 4*A*). SIRT5 knockdown can elevate succinylated levels of TCA-related metabolic enzymes (MDH2, SDHA, and OGDH), leading to their inactivity (25) (undefined). Hypersuccinylation of PDHA1 and CS has been proved to inhibit their enzymatic activities (26). We found the protein abundance of rate-limiting enzymes OGDH and IDH1 were decreasing at 24 hpi (Fig. 4*B*).

The main carbon sources of the TCA cycle include glucose and glutamine (27). We found glutaminase K164 was significantly succinylated after SARS-CoV-2 infection, while glutaminase abundance gradually decreased. Succinylation of K164 is able to promote ubiquitination of glutaminase K158 (28); therefore, the decreased utilization of glutamine in SARS-CoV-2–infected cells may be due to the succinylation of glutaminase K164, leading to its ubiquitinated degradation after viral infection. We also found eight succinylated proteins involved in glycolysis, as well as three proteins separately involved in fatty acid oxidation and mitochondrial transport. It was likely that succinylation may inhibit multiple metabolic enzymes, resulting in suppression of the TCA cycle.

### Succinylation Is Regulated by Viral Nonstructural Protein 14 through Interaction with Sirtuin 5.

**Fig. 5.**
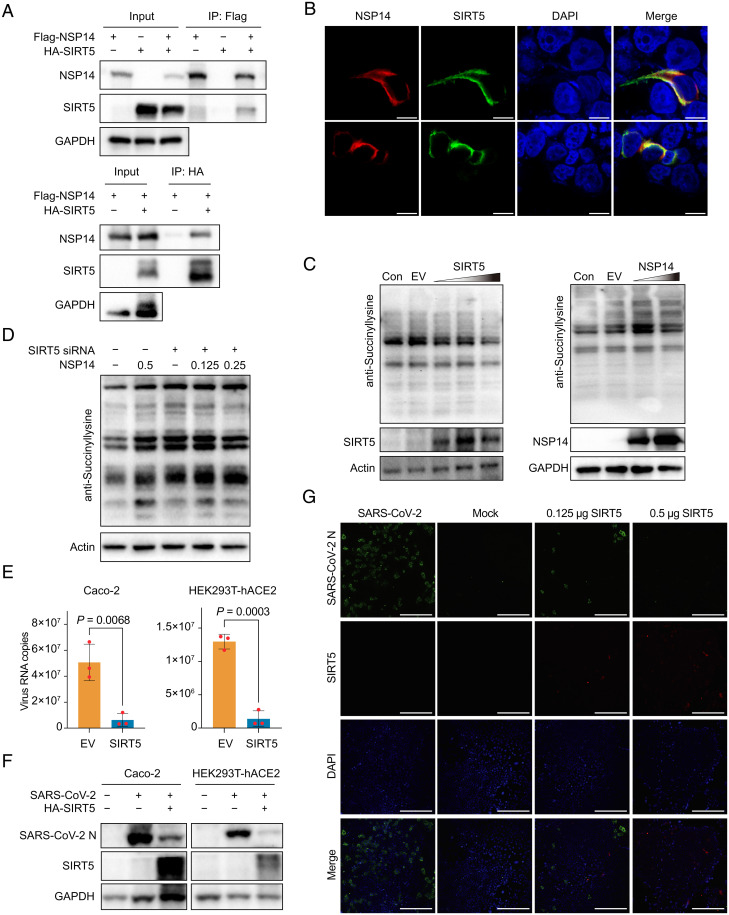
Regulation of protein succinylation and its effects on virus proliferation. (*A*) Co-IP analysis of NSP14 and SIRT5 in HEK293T cells. Vector Flag-NSP14 or HA-SIRT5 was transfected into HEK293T cells, and anti-Flag and HA IPs were analyzed by immunoblot with HA or Flag antibody. (*B*) Confocal imaging of NSP14 (red) and SIRT5 (green) in HEK293T cells. (Scale bars, 10 µm.) (*C*) Western blot analysis of pan-succinyllysine in HEK293T cells. Vector Flag-NSP14 or HA-SIRT5 with increasing amounts was transfected into HEK293T cells, following by the Western blot analysis with corresponding antibody. EV, empty vector; Con, control. (*D*) The succinylation levels after knockdown of SIRT5 by siRNA or overexpressed NSP14 were detected by the RT-qPCR in HEK293T cells. (*E*–*G*) Overexpression of SIRT5 reduced virus replication. Caco-2 and HEK293T-hACE2 cells were transfected with HA-SIRT5 vector, and infected with into SARS-CoV-2 after 24 h, then the viral replication was detected by the RT-qPCR (*E*) and Western blot (*F*) at 24 hpi. Student’s *t* tests were used for statistical analysis and the results are shown as smean ± SEM. Each experiment has three biological samples. (*G*) SIRT5 reduced SARS-CoV-2 proliferation in Caco-2 cells by IFA. After transfection of vector HA-SIRT5, viral N protein (green) and SIRT5 (red) were detected, nucleic DNA was stained by DAPI. (Scale bars, 100 µm.)

To determine the potential regulatory mechanism of host protein sucinylation, we examined the interactome of SARS-CoV-2, and found that viral nonstructural protein 14 (NSP14) can interact with sirtuin 5 (SIRT5) (29, 30). Functionally, NSP14 has the activity of 3′ to 5′ exonuclease and RNA methyltransferase, and SIRT5 has desuccinylase activity to remove succinylation in the cytoplasm and mitochondria (29, 30). We confirmed the interaction between viral NSP14 and SIRT5 by coimmunoprecipitation (co-IP) (Fig. 5*A*). Furthermore, we uncovered that both NSP14 and SIRT5 colocalized in the cytoplasm by confocal microscopy (Fig. 5*B*). Additionally, overexpression of SIRT5 in HEK293T cells could reduce the total succinylation level in host cells, while overexpression of NSP14 increased the overall succinylation level in transfected cells (Fig. 5*C*). These results demonstrated that SARS-CoV-2 NSP14 was able to promote host protein succinylation through interaction with SIRT5. Furthermore, knockdown of SIRT5 in HEK293T cells could significant up-regulate the overall level of succinylation in host cells (Fig. 5*D* and *SI Appendix,* Fig. S12). However, overexpressing NSP14 was not able to induce significant differences of the host succinylation levels (Fig. 5D).

We also investigated if host protein succinylation affects virus proliferation in vitro. Results showed that the suppression host protein succinylation induced by SIRT5 could effectively inhibit viral replication in both Caco-2 and HEK293T-hACE2 cells (Fig. 5 *E–G* and *SI Appendix*, Fig. S13). We also revealed that the NSP14 protein sequences of SARS-CoV-2 and other variants were similar (*SI Appendix*, Fig. S14), meaning that NSP14 from other variants might also promote the host protein succinylation by interacting with SIRT5.

### Antiviral Effects of the Succinylation Inhibitors.

**Fig. 6.**
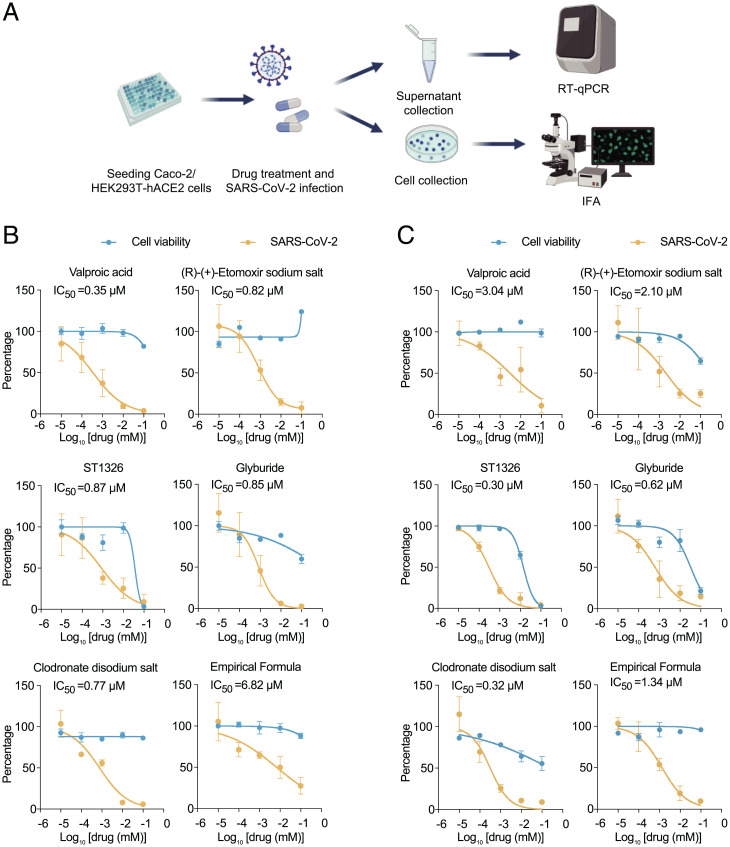
Inhibitors of succinylation in host cells reduce virus replication of SARS-CoV-2. (*A*) Schematic of viral infectivity and detection assays. Caco-2 and 293T-hACE2 cells were infected with SARS-CoV-2 (MOI = 0.01) and treated with 10 μM different drugs, and virus replication was detected by RT-qPCR and IFA. The detailed data of drugs used in the study are shown in *SI Appendix*, Table S1. (*B* and *C*) Cell viability was detected by cell counting (CCK-8) assay in Caco-2 (*B*) and 293T-hACE2 cells (*C*). Orange, viral infectivity; blue, cell viability. Independent experiments were repeated three times. Error bars represent the mean ± SEM of three biological replicates.

Several potential succinylation inhibitors targeting the succinylase, desuccinylase, or high-succinylated protein were selected to evaluate antiviral efficiencies in Caco-2 and HEK293T-hACE2 cells (Dataset S8). Carnitine palmitoyltransferase 1A (CAPT1A) and SIRT5 have the biological functions of succinylase and desuccinylase, respectively, and increased peroxisome proliferator-activated receptor-γ coactivator 1-α (PGC1-α) or inhibited AMPK can improve SIRT5 activity (31, 32). Therefore, we employed several pharmacological inhibitors of CPT1A and AMPK, and activators of PGC1-α to test their antiviral efficacy. Cells were infected with SARS-CoV-2 (MOI = 0.01) and treated with different concentrations of these drugs (Fig. 6*A*), the virus quantity and cell viability were quantified at 48 hpi.

As a result, 12 of these compounds were able to cause a strong inhibition of SARS-CoV-2 protein production. ST1326, an inhibitor of CPT1A, showed antiviral activity (IC_50_ = 0.86 μM) in Caco-2 cells and (IC_50_ = 0.541 μM) in HEK293T-ACE2 cells. The AMPK inhibitor STO-609 had obvious antiviral effect in Caco-2 cells with IC_50_ = 0.346 μM, but less antiviral activity in HEK293T-ACE2 cells (IC_50_ = 50.43 μM). The PGC1-α activator, valproic acid (VPA), showed antiviral activity in both cell lines, with an IC_50_ = 0.347 μM in Caco-2 cells and IC_50_ = 3.035 μM in HEK293T-ACE2 cells, respectively. Above all, there were six drugs showing strong antiviral activities against SARS-CoV-2 without affecting cell viability (Fig. 6 *B* and *C*).

SLC25A5 was the most succinylated protein during viral infection (Fig. 1*D*), whose specific inhibitors clodronate disodium salt and Empirical Formula (Hill Notation) could significantly repress SARS-CoV-2 replication in both two cell lines. The IC_50_ of clodronate disodium salt and Empirical Formula are 0.771 μM and 6.818 μM in Caco-2 cells, 0.317 and 0.6977 in HEK293T-hACE2 cells, respectively (Fig. 6 *B* and *C*).

Finally, immunofluroesence assay (IFA) was performed for further testing these drugs, (SI) > 40 and IC_50_ < 1 mentioned above, via two cell lines. Not unexpectedly, we found that VPA, ST1326, glyburide, and clodronic acid disodium salt all showed strong antiviral activity without obvious cytotoxicity (*SI Appendix*, Figs. S15–S17 and Table S1).

## Discussion

We used a mass spectrometry (MS)-based technology to investigate changes in protein abundance and succinylation during SARS-CoV-2 infection. Virus proteins were increased 12 hpi, suggestive of viral replication, whereas host proteins in abundance were decreased within 24 h. In contrast, pronounced protein succinylation changes were observed during the virus infection, highlighting that the degree by which the virus exploits host PTM to change the cellular signaling required for its replication.

Lysine succinylation is one of an identified type of PTMs involved in regulating metabolic processes, immunity, and inflammation (11, 33). For example, protein succinylation can alter the activity of the enzymes associated with glucose and lipid metabolisms (34, 35); desuccinylation suppresses activation of the signaling adaptor MAVS, thereby inhibiting antiviral gene expression and type I interferon production (36). The Toll-like receptor 4 signaling pathway affects the succinylation processes and pathways (37). In this study, SARS-CoV-2 infection was shown to promote succinylation of key enzymes for the TCA cycle, glycolysis, fatty acid oxidation, and mitochondrial transport, but expression of these enzymes, especially the rate-limiting enzymes OGDH and IDH1, was usually decreased, whereby inhibiting the cellular metabolic pathways, which may contribute to virus replication.

Protein succinylation is regulated by reversible succinylase and desuccinylase. So far, KAT2A and CPT1A have been confirmed to have a succinylase activity, and SIRT5 has a desuccinylase activity. However, KAT2A acts as a histone-specific succinylase responsible for succinylation of histone (31). We identified a regulatory mechanism of host protein succinylation, which is mediated by SARS-CoV-2 NSP14 through interaction with SIRT5.

As inhibition of host protein succinylation can effectively suppress virus replication in infected cells, SIRT5 activators and CPT1A inhibitors may possess antiviral activity. The activator of SIRT5 remains to be identified, but it is regulated by PGC1-α and AMPK (32). PGC1-α activator VPA, AMPK inhibitors GSK690693, and STO-609 may increase desuccinylase activity of SIRT5. VPA, a histone deacetylase inhibitor, is clinically used as a broad-spectrum antiepileptic drug. VPA can up-regulate the transcriptional coactivator PGC1-α that further activates SIRT5 (32, 38). Additionally, VPA can reduce ACE-2 expression, and modulate production of inflammatory cytokines, supporting that it is a promising antiviral drug for COVID-19 (39). GSK690693, a potent pan-AKT kinase inhibitor, is currently in development for clinical treatment of various cancers (40). STO-609, an inhibitor of calmodulin-dependent protein kinase kinase (CaM-KK), can impair AMPK phosphorylation (41). CPT1A inhibitors ST1326, glyburide, and etomoxir had different antiviral effects, which may be associated with different functional targets; For example, ST1326 is a fatty acid oxidation inhibitor, glyburide is a KATP channel antagonist, while etomoxir is an irreversible inhibitor of CPT1A. The antiviral activity of these compounds or drugs suggests that host protein succinylation may become potential drug targets against SARS-CoV-2. Although we did not perform the related antiviral detection on Delta, Omicron, or Lambda variants, in which high similarities of NSP14 were found, we speculate that these drugs may also have similar antiviral effects.

In summary, viral rapid evolution and emerging variants have concern for enhanced transmission, immune evasion, or enhanced virulence of SARS-CoV-2, highlighting development of host-directed broad-spectrum antiviral drugs. The global succinylproteomics analysis here revealed the host cellular metabolic processes hijacked by SARS-CoV-2 and viral protein succinylation modification. We identified several succinylation-targeted compounds or drugs that can effectively inhibit SARS-CoV-2 replication in cells. We hope that this work will further elucidate the pathogenesis of SARS-CoV-2 and accelerate the discovery of host-directed broad-spectrum antiviral therapies for COVID-19.

## Materials and Methods

### Cells, Virus, and Plasmids.

Human colorectal adenocarcinoma epithelial cell Caco-2, human kidney epithelial cell derived HEK293T-hACE2, and African green monkey kidney epithelial cell Vero E6 were maintained in Dulbecco’s modified Eagle’s medium (DMEM) containing 1% penicillin-streptomycin and 10% FBS (Gibco). The HEK293T-hACE2 cell was kindly provided by Zhong Ji Dang Kang Biotechnology Co., Ltd., Beijing, China. All cell lines were cultured at 37 °C with 5% CO_2_. SARS-CoV-2 isolate CHN/Beijing_IME-BJ01/2020 (GenBank accession no. MT291831.1), originated from human throat swabs, was propagated in Vero E6 cells in DMEM (HyClone) supplemented with 2% FBS, and titred using a tissue culture median infectious dose assay (TCID_50_). All experiments of infectious SARS-CoV-2 were conducted under biosafety level 3 facilities. HA-tagged SIRT5 plasmid was purchased from Miaoling Biotech, and the SARS-CoV NSP14 was cloned into vector VR1012 with the Flag-tag (Sangon Biotech) and confirmed by sequencing.

### Cell Viability Assays.

Caco-2 or HEK293T-hACE2 cells were plated into 96-well plates and leave adherence for 24 h. The culture medium was then discarded, and SARS-CoV-2 virus (MOI = 0.01, 0.1, and 1, respectively) or drugs/compounds (0 to 100 μM) mixed in 100 μL DMEM containing 2% FBS were added into the plates. After incubation for 48 h, 10 μL of CCK8 solution (Dojindo Laboratories) was added to each well; the OD value at 450 nm was measured after another 2 h of incubation (42).

### Cell Infection for Proteomics and Transcriptomics.

Caco-2 cells (2 × 10^6^ cells) were seeded in 25-cm^2^ tissue-culture flasks (Corning). The following day, cells were inoculated with SARS-CoV-2 at an MOI of 0.01 in serum-free DMEM for 1 h at 37 °C. After absorption, the 0-h samples were harvested immediately, and the media of other samples were replaced with fresh 2% FBS/DMEM and incubated for indicated time at 37 °C before lysis for further analysis.

### Immunoblot and Co-IP.

For detection of protein PTMs, Caco-2 cells were harvested at 0, 12, and 24 hpi for immunoblot analysis. Proteins were quantified using Pierce Gold BCA (ThermoFisher). Total protein (20 μg) from lysed cells was separated by 12% SDS/PAGE using Tris-Glycine-SDS running buffer (Yeasen Biotech). The proteins were then transferred on a PVDF membrane (Millipore) in transfer buffer (20% methanol, 25 mM glycine, and 25 mM Tris). The membranes were blocked by 5% BSA (Sigma) for 1 h at room temperature, and incubated at 4 °C overnight with antiacetyllysine antibody (PTM Biolab; 1:1,000), antisuccinyllysine antibody (PTM Biolab; 1:1,000 dilution), antimalonyllysine antibody (PTM Biolab; 1:1,000), antiubiquitin antibody (PTM Biolab; 1:2,000), or antilactyllysine antibody (PTM Biolab; 1:1,000) as primary antibody, respectively. After washing three times with PBST (PBS+0.5% Tween 20), the membranes were detected by goat anti-rabbit or mouse IgG (H+L) (Invitrogen; 1:10,000) by incubation for 1.5 h at room temperature. Chemi-luminescence was measured with ECL (ThermoFisher) and protein bands were visualized under Biorad CHemiDoc XRS (Bio-Rad).

Co-IP was conducted as described elsewhere (43). Briefly, 2 μg of HA-SIRT5 or Flag-NSP14 plasmids were transfected into 2 × 10^6^ HEK293T cells in a six-well plate using Lipo 3000 (Invitrogen) according to the manufacturer’s recommendations; cells were harvested after 30 h and lysed by 500 μL lysis buffer (50 mM Tris⋅HCl, pH 8.0, 150 mM NaCl, 1% Nonidet P-40) supplemented with protease and phosphatase inhibitors (Selleck). Ten percent of the cell lysates were separated as an input and the remaining lysates were incubated with 10 μL anti-HA or anti-Flag agarose (Sigma) on a roller at 4 °C overnight. The lysates were washed five times with 1 mL lysis buffer and boiled at 100 °C for 10 min, and the concentrated supernatant were saved as IPs. Cell lysates and IPs were analyzed by immunoblot assay using anti-HA and anti-Flag antibodies (Proteintech).

### MS and Data Processing.

The infected cells were collected at 0, 12, and 24 hpi, respectively, and lysed by ultrasonication. Equal amounts of protein samples were acetone precipitated, and subjected to trypsin digestion for MS analysis to determine both total protein abundance and succinylation.

Affinity enrichment of succinylated peptides and liquid-chromatography MS of proteome and succinyl-proteome was conducted by PTM BioLab in Hangzhou, China. Briefly, peptides of each sample were separated by nanoElute (Bruker), and all MS spectra were collected in the timsTOF Pro (Bruker) with parallel accumulation-serial fragmentation (PASEF) acquisition mode.

Raw MS data of proteomics and succinyl-proteomics and protein identification were performed by MaxQuant (v1.6.17.0) with default values and label-free quantification enabled, as described previously (44). All MS data were searched against the protein databases of uniprot-filtered human sequences (20,407 proteins, downloaded on May 25, 2021), uniprot-reviewed mycoplasma sequences (3,040 proteins, downloaded on May 25, 2021), and SARS-Cov-2 (GenBank accession no. MT291831.1) to identify protein sequences. For protein abundance analysis, data were searched using the default andromeda settings, with variable modification of methionine oxidation and static modification of carbamidomethyl cysteine. For succinylpeptide enriched analysis, andromeda settings were modified to include succinylation of lysine as a variable modification.

### Total RNA-Sequencing and Data Processing.

Library preparation and transcriptome sequencing was conducted by Guangzhou Gene Denovo Biotechnology Co. Ltd. Briefly, total RNA was extracted by TRIzol (Invitrogen). Ribo- and ploy(A)^+^ RNA-seq libraries were prepared according to standard protocol (45). Libraries were sequenced with Illumina HiSeq 4000 using a 2 × 150 paired-end sequencing strategy. The clean data were matched to the human genome (GRCh38) and SARS-CoV-2 genome (MT291831.1) using STAR (2.7.7a) (46). Transcripts were quantified and annotated with feature Counts (v1.6.0). All transcripts were normalized and differentially analyzed in DESeq2 (47).

### Sequence Alignment Analysis of Homolog Proteins.

Sequences of M, N, and NSP14 proteins of SARS-CoV-2 (MT291831.1) and other coronaviruses (Bat SARS-like coronavirus isolate Rs4231: KY417146.1, MERS-CoV: JX869059, SARS coronavirus Urbani: AY278741.1, SARS-CoV Alpha: B.1.1.7, SARS-CoV Beta: B.1.351, SARS-CoV Gamma: B.1.1.28.1, SARS-CoV Delta: B.1.617.2, SARS-CoV Lambda: C.37, SARS-CoV Omicron: B.1.1.529) were obtained from the National Center for Biotechnology Information (NCBI) GenBank. Multiple sequences alignment analysis was performed with CLUSTALW (https://www.genome.jp/tools-bin/clustalw).

### Bioinformatics Analysis.

We used MSstats to identify significantly expressed proteins and succinylated sites in infected cells at different time points (48). By default, intensity data of proteins or succinylated sites were processed using MSstats with default values. We used Student’s *t* test to calculate *P* value, and the Benjamini–Hochberg method was used to correct the *P* value. We performed enrichment analysis of gene ontology and Kyoto Encyclopedia of Genes and Genomes pathways via ClueGo (49) or clusterProfiler package in R (4.0.4) with default parameters (50).

### Viral Inhibitors Assay.

Caco-2 and HEK293T-hACE2 cells were seeded in 96-well plates at 5,000 cells per well. Once the cells grow adhering to the wall, the medium was replaced with 100 μL of fresh medium containing either 10 nM, 100 nM, 1 μM, 10 μM, 100 μM of drugs or DMSO as a control, and infection was conducted by adding SARS-CoV-2 (MOI of 0.01). There were three biological replicates of each drug. The plates were incubated for another 48 h and supernatants were collected for virus quantification by quantitative reverse-transcription PCR (RT-qPCR). Detailed information of the drugs and compounds is provided in Dataset S8.

### Immunofluorescence.

Caco-2 and HEK293T-hACE2 cells were cultured on coverslips in 24-well plates. After the viral infection and drug treatment for 48 h, as described above, cells were fixed with 4% paraformaldehyde (Biotopped) at dark for 30 min, the cells were then carefully washed, and permeated with 0.5% Triton X-100 (Yeasen Biotech) in PBS for 15 min. Cells were blocked in 1% BSA at room temperature for 1 h, and stained with the monoclonal antibody of anti–SARS-CoV-2 nucleocapsid protein (N protein) (CSB-DA701BmN, Wuhan Huamei Biotech) at 4 °C overnight. The cells were then washed three times with PBS+0.1%BSA, and incubated with CoraLite 594- and CoraLite 488-conjugated IgG secondary antibodies (Proteintech). Nuclei were stained with DAPI (Yesen Biotechnology). Fluorescence images were analyzed with a fluorescence microscope (Nikon) or a confocal microscope (FV3000, Olympus).

### RT-qPCR.

RNA from the supernatant of infected host cells was extracted by the UNlQ-10 Column TRIzol total RNA isolation kit (Sangon Biotech Shanghai), and cDNA was synthesized with TransScript One-Step gDNA Removal and cDNA Synthesis SuperMix (TransGen). Virus copies were determined by the HiScript II One Step qRT-PCR SYBR Green Kit (Vazyme), as described previously (42).

### Statistical Analysis.

We did not predetermine the sample size using statistical methods. Significance was determined using the unpaired, two-sided Student’s *t* tests unless otherwise stated. Statistical analysis was performed using GraphPad Prism 8. For clustering and pathway enrichment analysis, the corresponding R packages were used for all statistical tests.

## Supplementary Material

Supplementary File

Supplementary File

Supplementary File

Supplementary File

Supplementary File

Supplementary File

Supplementary File

Supplementary File

Supplementary File

## Data Availability

All mass spectrometry data were uploaded to iProX (IPX0003647000), and the raw transcriptome data were uploaded to the NCBI Sequence Read Archive, https://www.ncbi.nlm.nih.gov/sra (accession no. PRJNA783650) (53) .All other study data are included in the main text and/or supporting information.
