# Supplementary material for: The global succinylation of SARS-CoV-2–infected host cells reveals drug targets
Source: Proc Natl Acad Sci U S A. 2022 Jul 12;119(30):e2123065119. doi: 10.1073/pnas.2123065119 (PMC9335334; doi:10.1073/pnas.2123065119)
Supplement: Supplementary File [file pnas.2123065119.sapp.pdf]

Supplementary Information for
The global succinylation of SARS-CoV-2-infected host cells reveals drug targets

Quan Liu, Heming Wang, He Zhang, Liyan Sui, Letian Li, Wang Xu, Shouwen Du, Pengfei Hao, Yuhang Jiang, Jing Chen, Xiaoyun Qu, Mingyao Tian, Yinghua Zhao, Xuerui Guo, Xingye Wang, Wu Song, Guangqi Song, Zhengkai Wei, Zhijun Hou, Guoqing Wang, Minhua Sun, Xiao Li, Huijun Lu, Xinyu Zhuang, Ningyi Jin, Yicheng Zhao, Chang Li, Ming Liao

Co-corresponding authors: Ningyi Jin, Yicheng Zhao, Chang Li, and Ming Liao
Email: ningyik@126.com; yichengzhao@live.cn; lichang78@163.com; mliao@scau.edu.cn.

This PDF file includes:

Figures S1 to S17
Table S1
Legends for Datasets S1 to S8
SI References

Other supplementary materials for this manuscript include the following:

Datasets S1 to S8

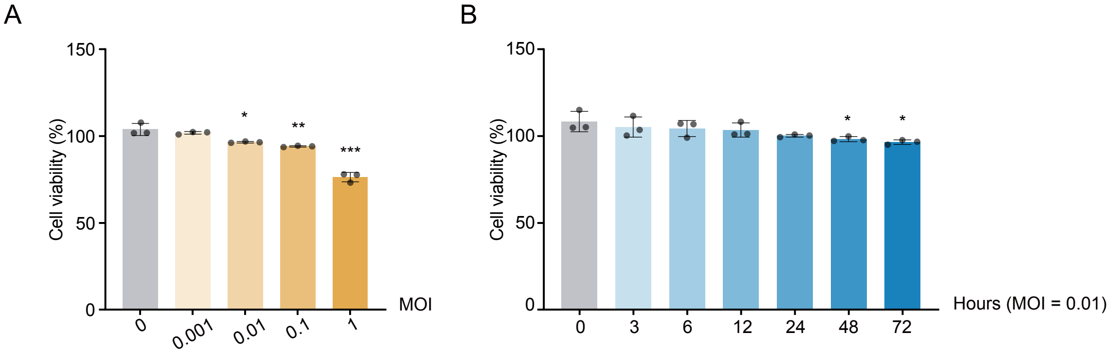

Fig. S1. Cell viability of SARS-CoV-2-infected cells. (A) Caco-2 cells were infected with SARS-CoV-2 at the MOI of 0, 0.001, 0.01, 0.1, and 1 for 48 h, and the cell viability was detected by a cell counting (CCK-8) assay. (B) Caco-2 cells were infected with the virus at an MOI of 0.01, and cell viability was determined at 0, 3, 6, 12, 24, 48, and 72 hpi using CCK-8 assay. Data for each condition were collected for three biological replicates. Student's *t* test was used for statistical analyses. *, $p < 0.05$; **, $p < 0.01$. MOI, multiplicity of infection.

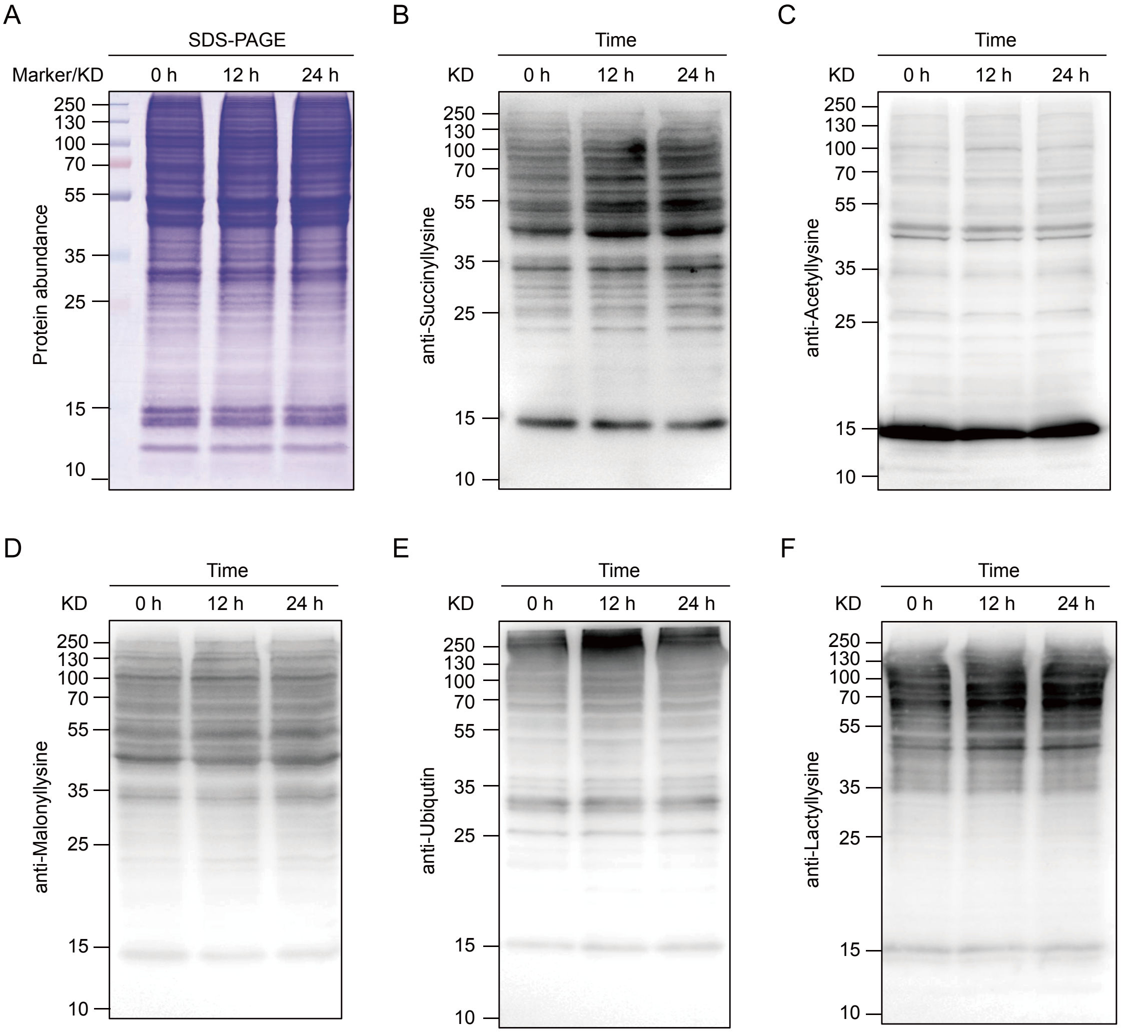

Fig. S2. Protein post-translational modifications in SARS-CoV-2-infected cells. Infected Caco-2 cells with SARS-CoV-2 (MOI=0.01) at 0, 12 and 24 h post infection were harvested for cell lysis. Total protein expression was analyzed by SDS-PAGE electrophoresis (A) and post-translatinal modifications were detected by Western blot for succinyllysine (B), acetyllysine (C), malonyllysine (D), ubiqutin (E), and lactyllysine (F).

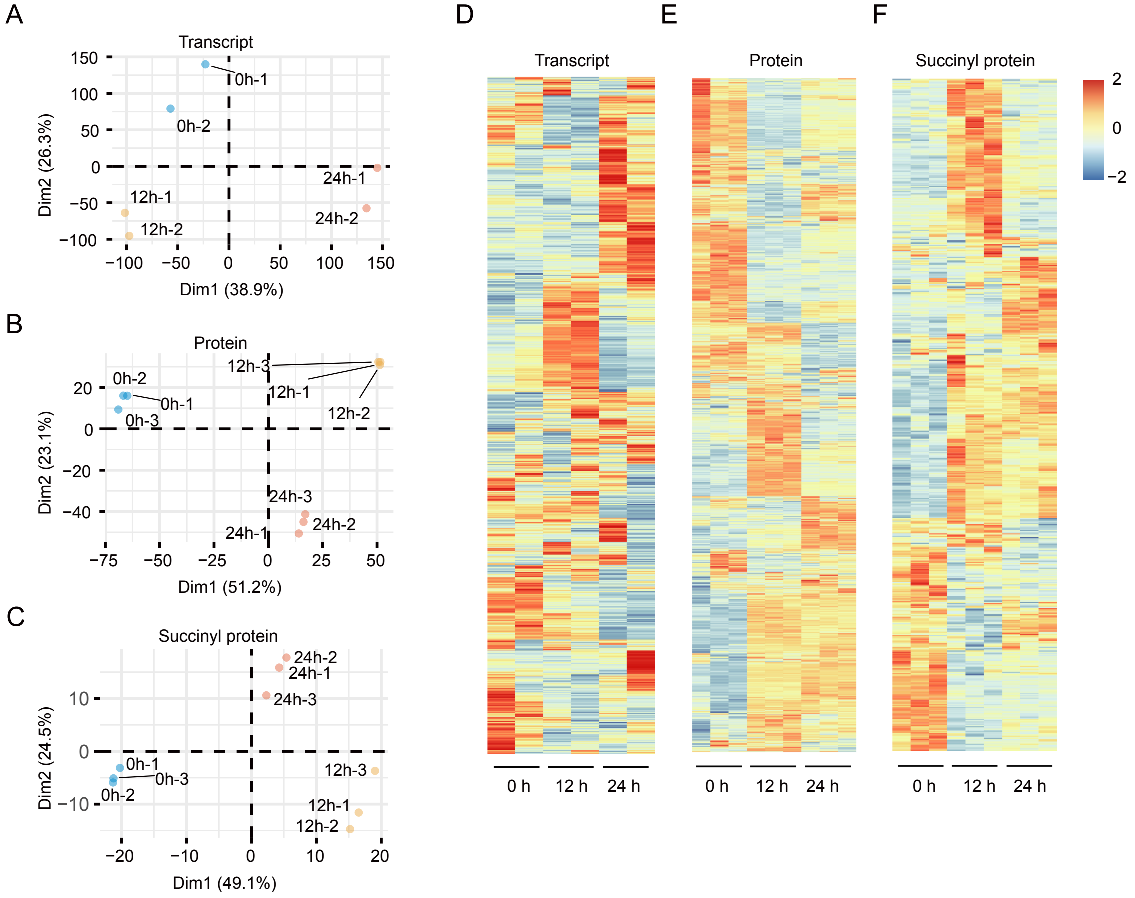

Fig. S3. Quality control of multi-omics data by principal component analysis and cluster analysis. (A-C) Principal component analysis (PCA) of transcriptomics (A), PCA of proteomics (B), PCA of succinyl-proteomics (C). PCA was conducted with the R package (factoextra). Sequencing reads or protein intensities were Log_2 transformed, followed by PCA and visualization. The colors represent the infected samples at different time points. (D-F) Heatmap of transcriptomics (D), Heatmap of proteomics (E), Heatmap of succinyl-proteomics (F). All quantified data were Log_2 transformed, and the Euclidean distance between different samples was calculated, followed by visualization. Each column represents a sample, and each row represents a transcript or protein.

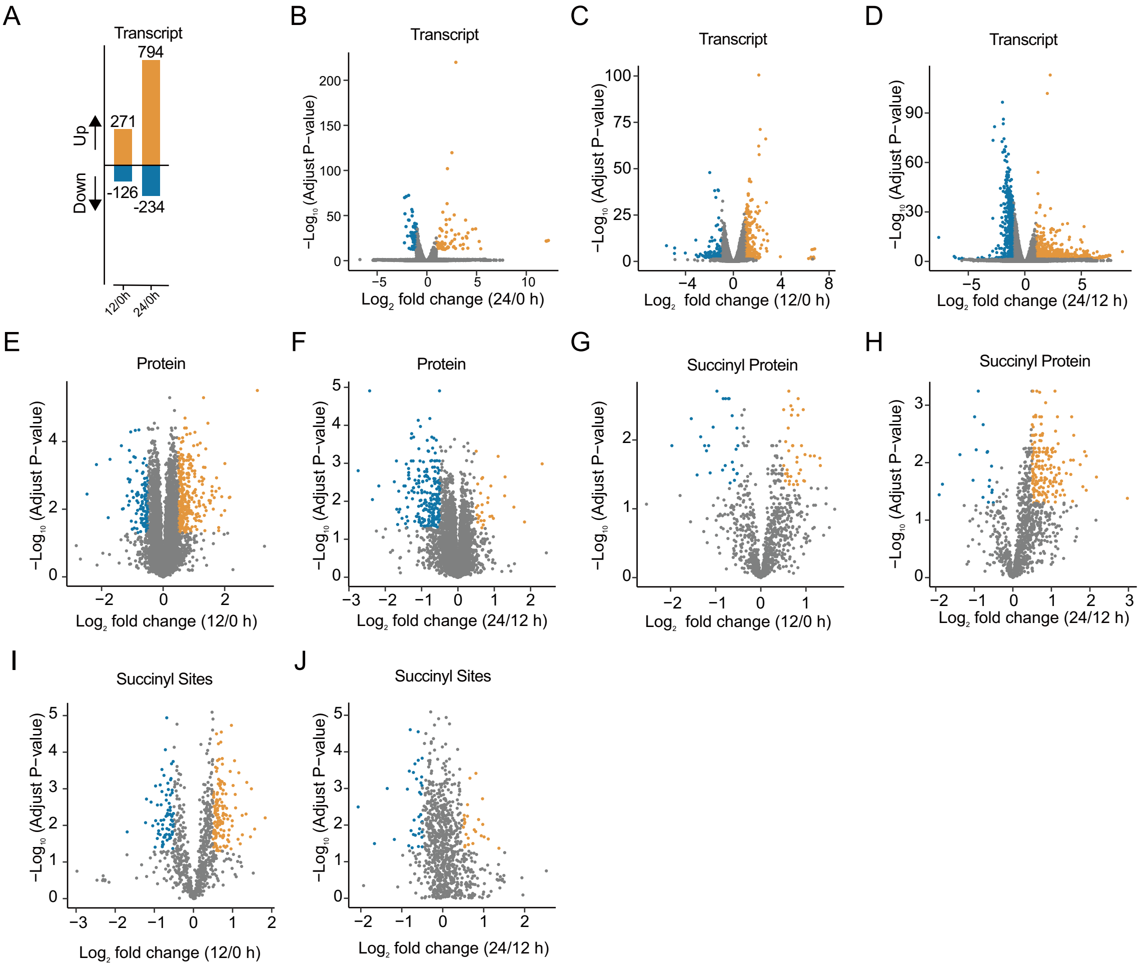

Fig. S4. Volcano plot of multi-omics data. (A-D) Transcript expression change in infected cells. The increased (orange) or decreased (blue) number of transcripts across the infection time course (A). Volcano plot of transcript expression in cells at 24 hpi as comparison with 0 hpi (B). Volcano plot of transcript expression in cells at 12 hpi as comparison with 0 hpi (C). Volcano plot of transcript expression in cells at 24 hpi as comparison with 12 hpi (D). (E-F) Protein abundance change in infected cells. Volcano plot of protein abundance quantification in cells at 12 hpi as comparison with 0 hpi (E). Volcano plot of protein abundance quantification in cells at 24 h post-infection as comparison with 12 h after infection (F). (G-H) Succinylated protein abundance change in infected cells. Volcano plot of succinylated protein abundance in cells at 12 hpi as comparison with 0 hpi (G). Volcano plot of succinylated protein abundance in cells at 24 hpi as comparison with 12 hpi (H). (I-J), Succinylated sites change in infected cells. Volcano plot of succinylated sites in cells at 12 hpi as comparison with 0 hpi (I). Volcano plot of succinylated sites in cells at 24 hpi as comparison with 12 hpi (J). We defined Log_2 fold change > 0.5 as up-regulated proteins/sites ($p < 0.01$), and Log_2 fold change < -0.5 as down-regulated proteins/sites ($p < 0.01$).

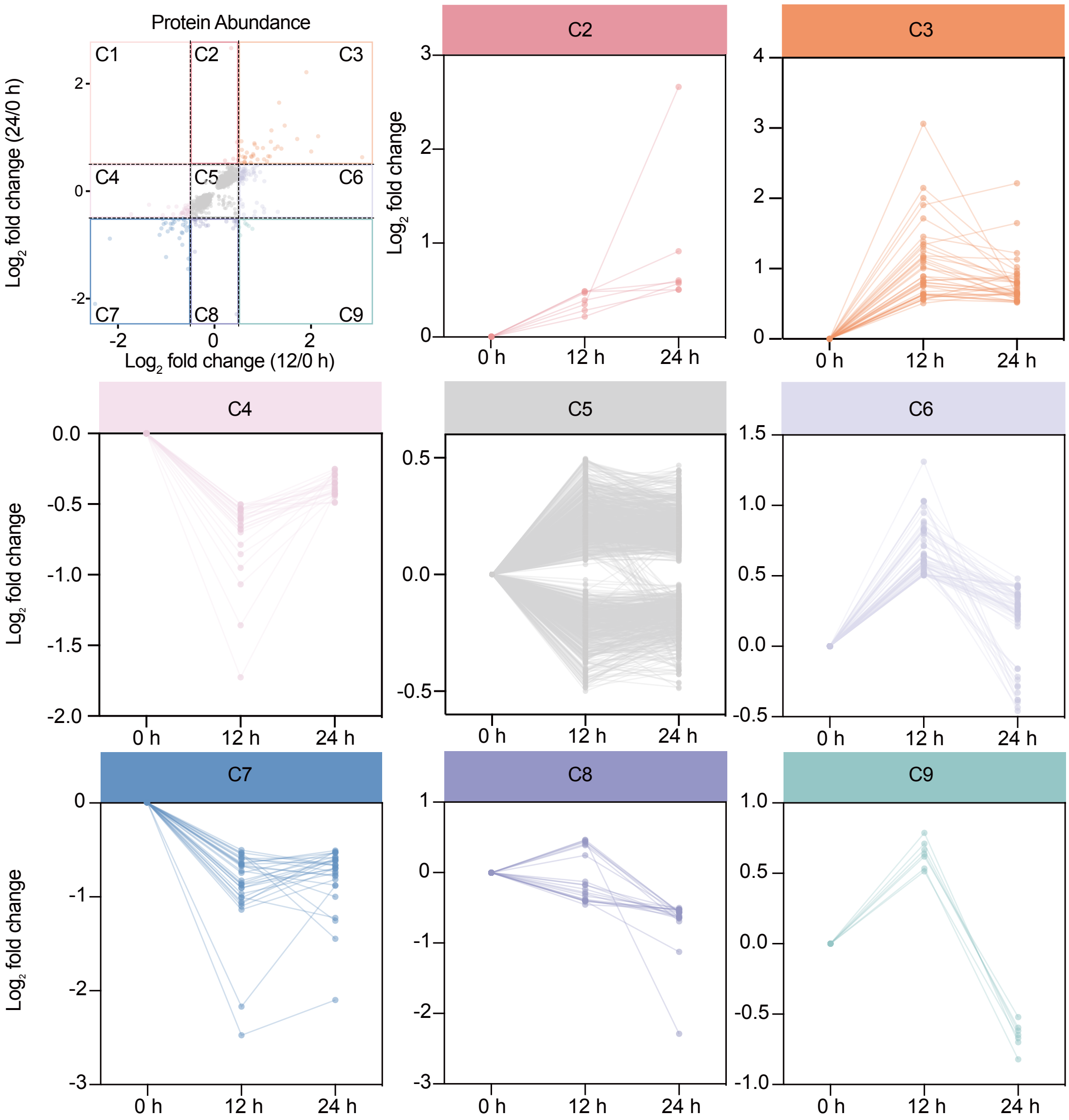

Fig. S5. Cluster analysis for changed host proteins in abundance by SARS-CoV-2. Scatterplot for fold change of host protein abundance in 12/0 h compared to 24/0 h, all host proteins were divided into 9 clusters based on the fold changes. The dashed line represents Log_2 fold change of ± 0.5. Scatterplot for the fold change of host proteins at 12 h and 24 h (compared with 0 h) in each cluster.

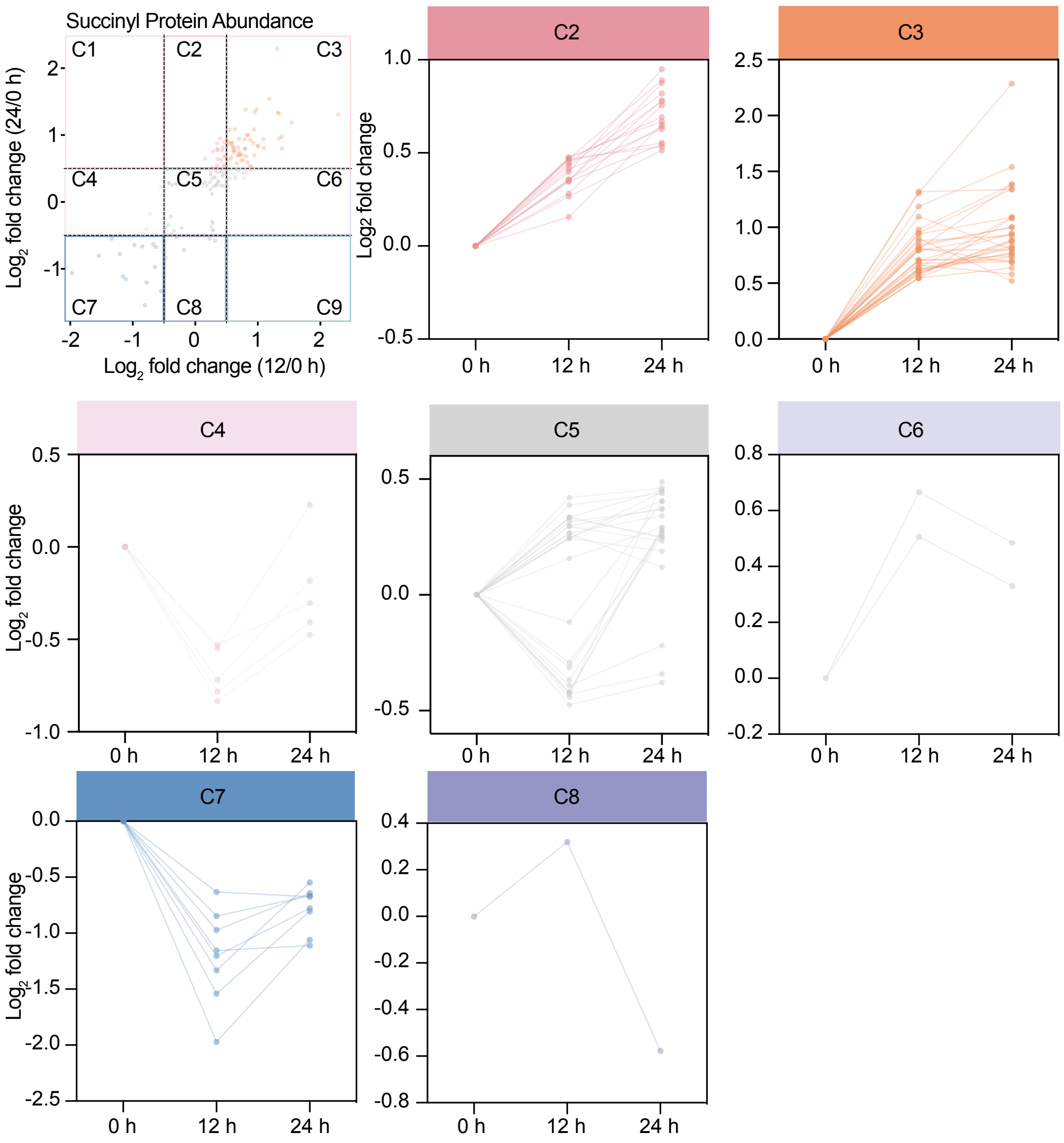

Fig. S6. Cluster analysis for changed host succinyl-protein in abundance by SARS-CoV-2. Scatterplot for fold change of host succinyl-proteins in 12/0 h compared to 24/0 h, all succinyl-proteins were divided into 7 clusters based on the fold changes. The dashed line represents Log_2 fold change of ± 0.5. Scatterplot for the fold change of succinyl-proteins at 12 h and 24 h (compared with 0 h) in each cluster.

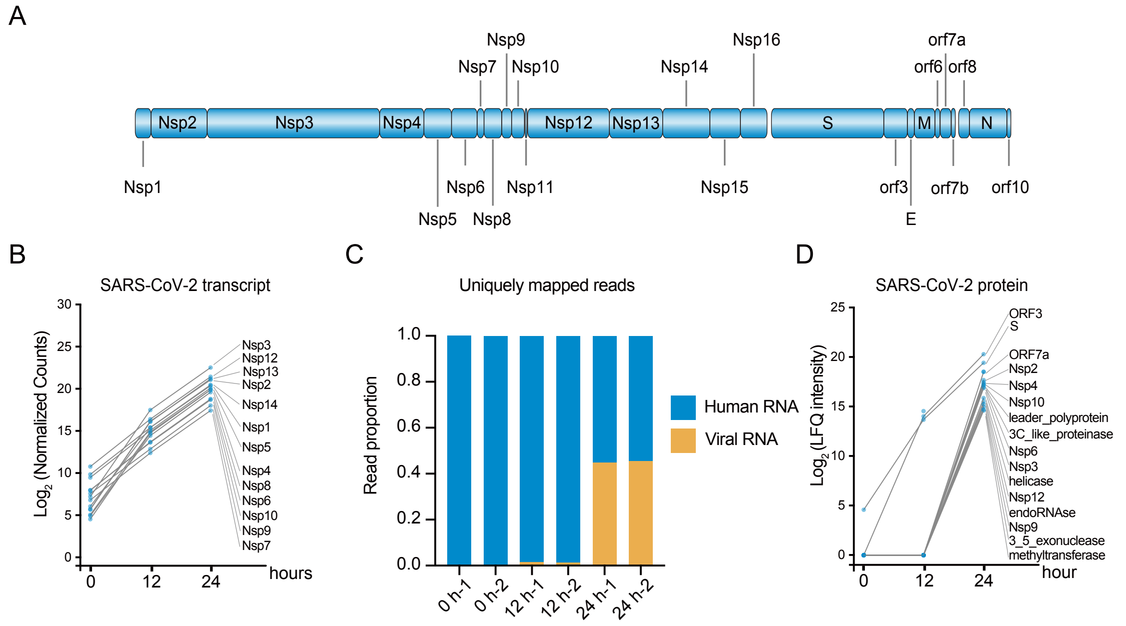

Fig. S7. Quantitative analysis of viral transcripts and proteins in SARS-CoV-2-infected cells. (A) The genome structure of SARS-CoV-2. The virus genome encodes 4 structural proteins (E, M, S, and N), 16 non-structural proteins (Nsp1-16), and 6 accessory factors (Orf3, Orf6, Orf7a, Orf7b, Orf8, and Orf10). (B) The viral transcripts of SARS-CoV-2 in infected cells. (C) Proportion of viral transcripts and host transcripts in SARS-CoV-2-infected cells, showing that the viral transcripts accounted for 40% of the cellular total transcripts at 24 h post infection. (D) Viral protein abundance of SARS-CoV-2-infected cells, with the exception of N and M proteins.

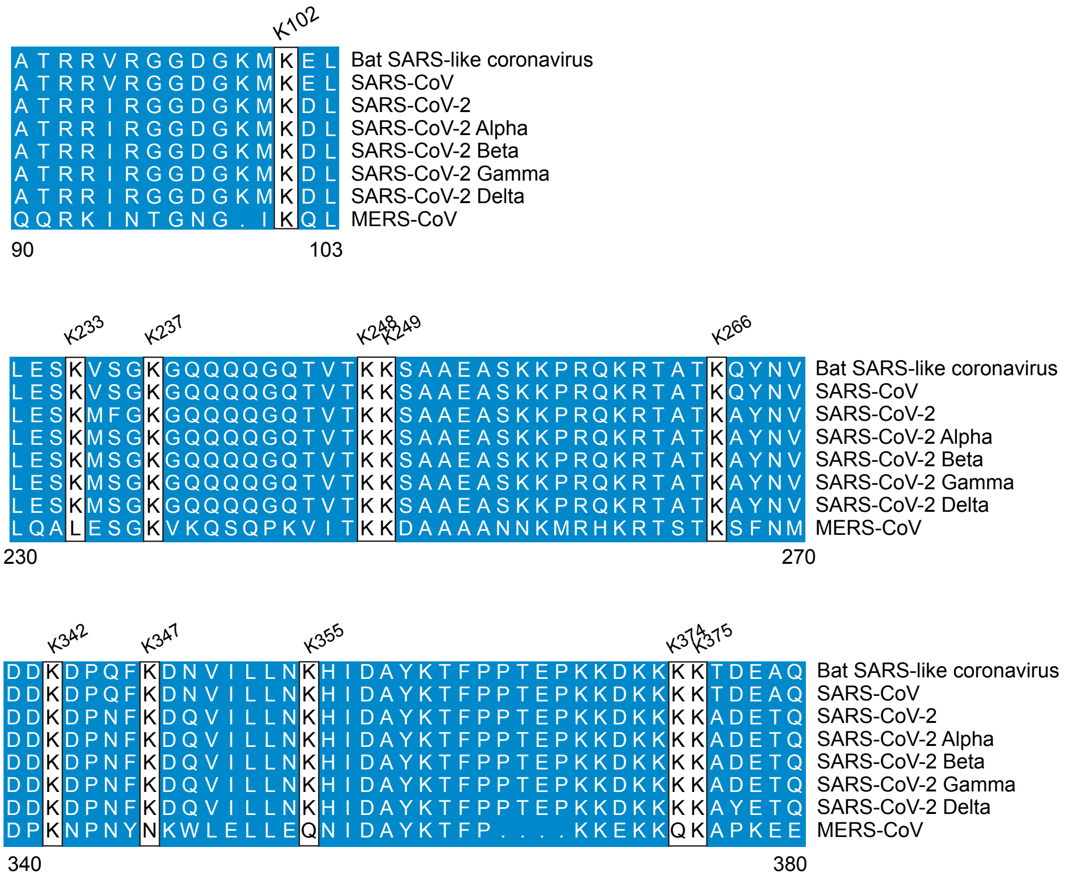

Fig. S8. Conserved succinylated sites in N protein of SARS-CoV-2 and other coronaviruses. Eleven conserved succinylated sites in N protein of coronaviruses are shown in the sequence alignment.

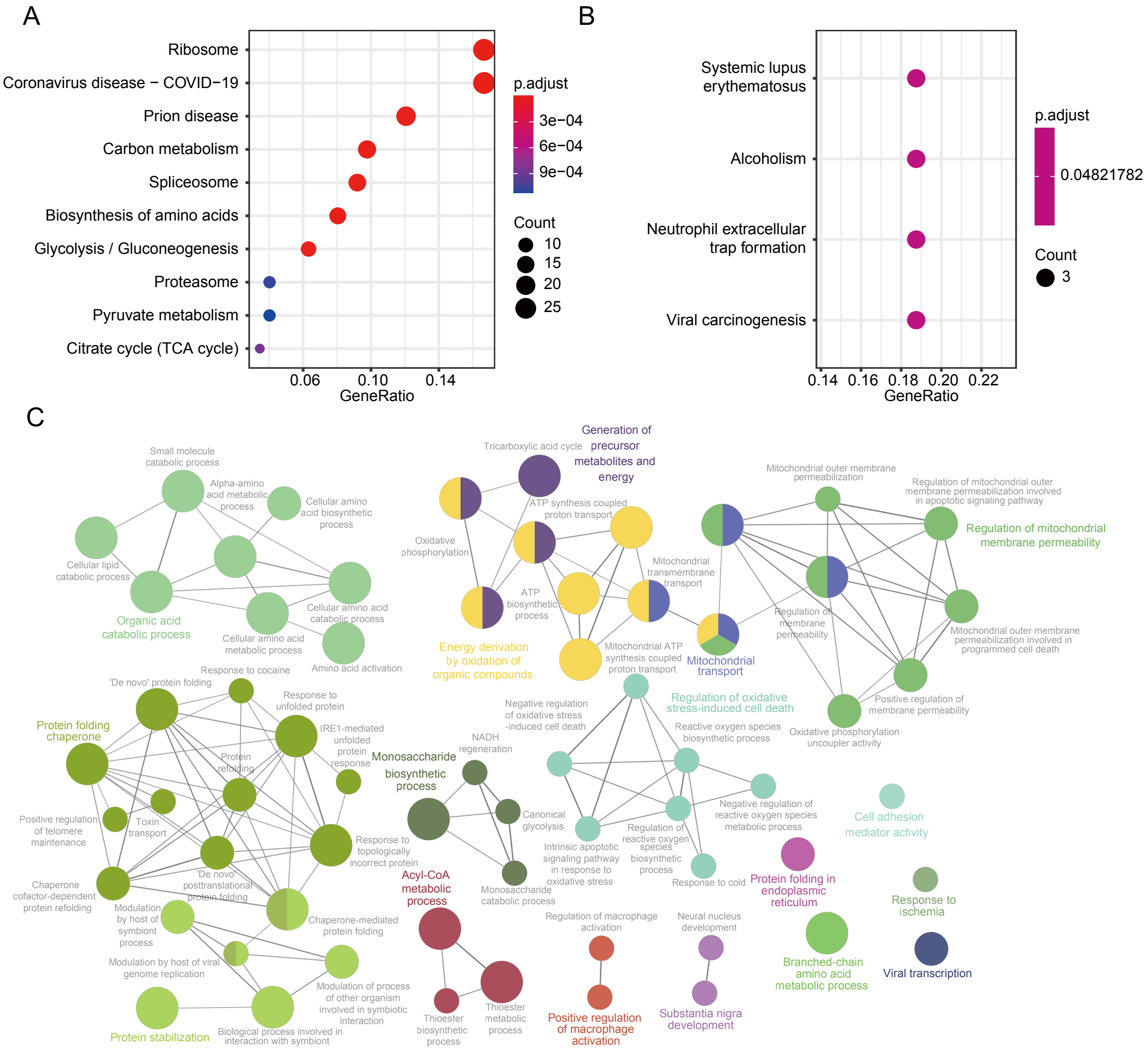

Fig. S9. Enrichment analysis for changed succinylated protein in infected cells. (A) KEGG enrichment analysis of up-regulated succinylated protein (Log_2 fold change > 0.5) in infected cells 24/0. (B) KEGG pathway enrichment analysis down-regulated succinylated protein (Log_2 fold change < -0.5) in infected cells 24/0 h. KEGG pathway enrichment was analyzed using the clusterProfiler (1). Depth of color indicates the significance of the term (adjusted *p*-value), the size indicates the gene count. (C) GO enrichment analysis for up-regulated protein succinylation (Log_2 fold change > 0.5).

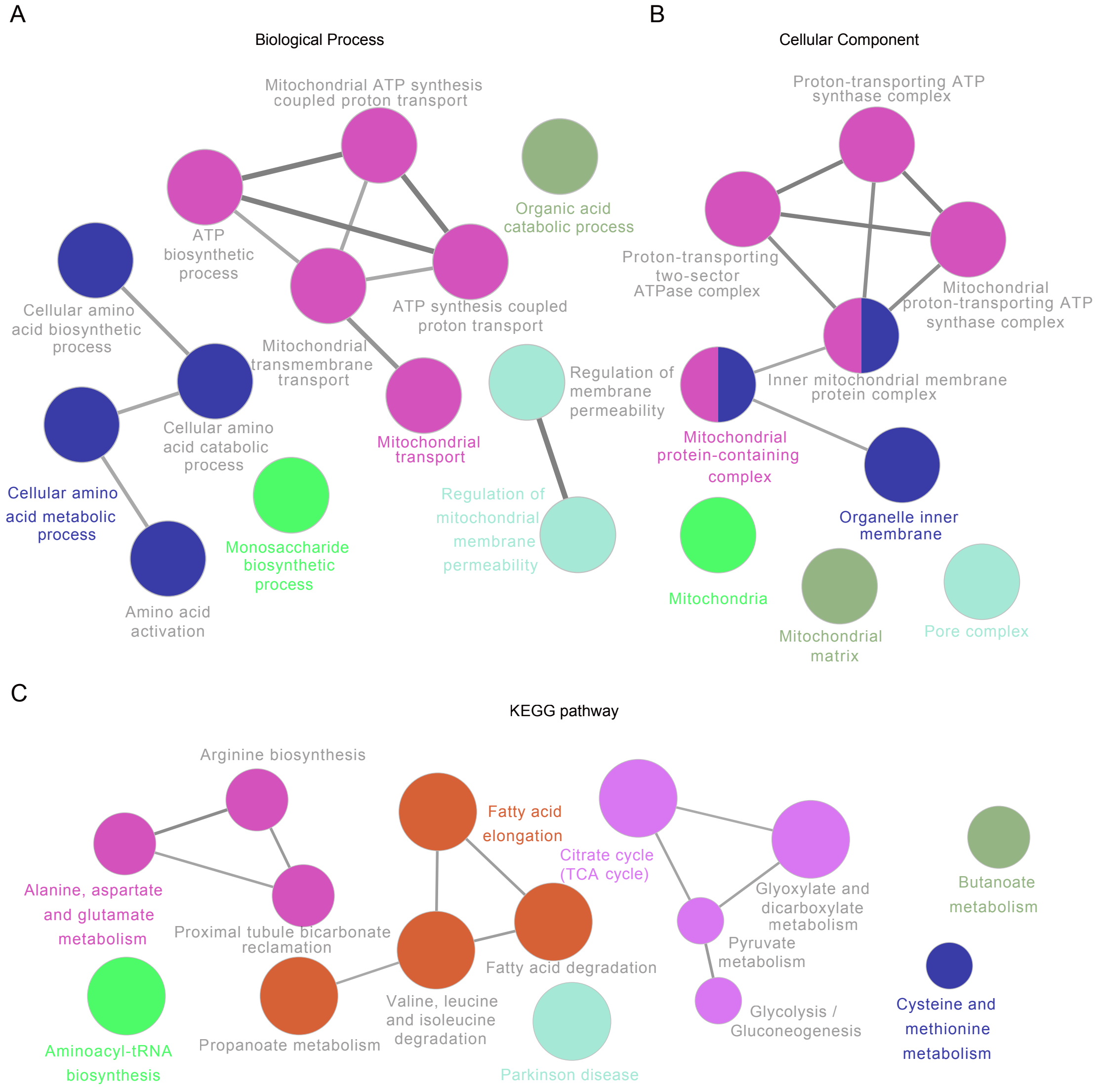

Fig. S10. Enrichment analysis for regulated succinylated sites in infected cells. (A-B) GO enrichment analysis of succinylated sites (Fig. 3*B* cluster 3) includes biological processes (A) and cellular components (B). (C) KEGG pathway enrichment analysis of succinylatd sites (Fig. 3*B* cluster 3). GO and KEGG pathway enrichment were analyzed using the ClueGo (https://apps.cytoscape.org/apps/cluego).

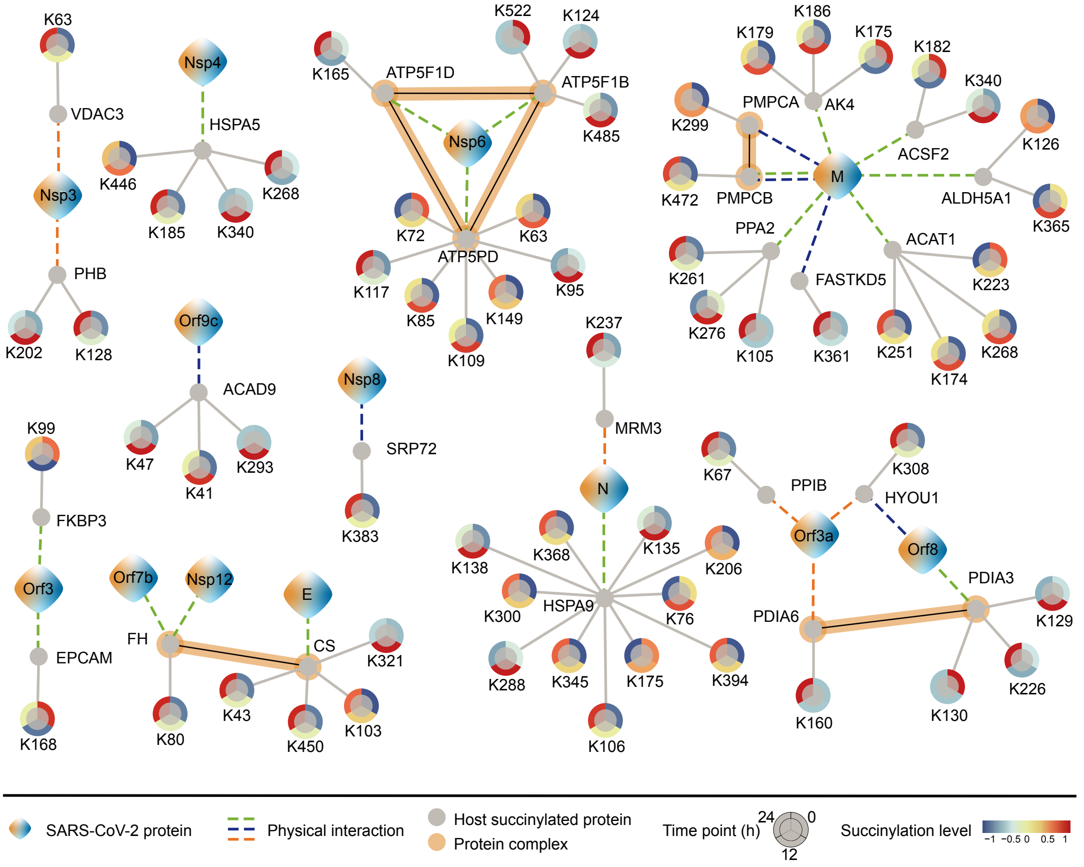

Figure S11. Interaction between viral proteins and succinylated host proteins in infected cells. The SARS-CoV-2 virus-host protein-protein interaction reveals 1,417 human proteins interacting with 26 viral proteins (2, 3, 4). Here we found 25 of 1,417 proteins were significantly differentially succinylated across the virus infection. Viral proteins are shown as rhombi. Interacting host proteins are shown as gray circles. Succinylated sites emanate from host proteins, colored by their z-score fold changes compared with 0-h infected samples (red, increase; blue, decrease) at each time point (0, 12, and 24 h after infection) in a clockwise fashion.

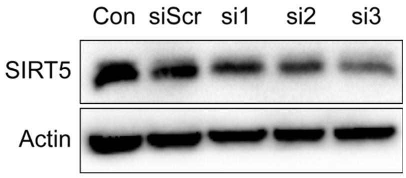

Fig. S12. Validation of the SIRT5 knockdown efficiency in HEK293T. siRNAs of SIRT5 were separately transfected into HEK293T cells, following by the Western blot analysis with corresponding antibody, Con: control, siScr: scrambled siRNA.

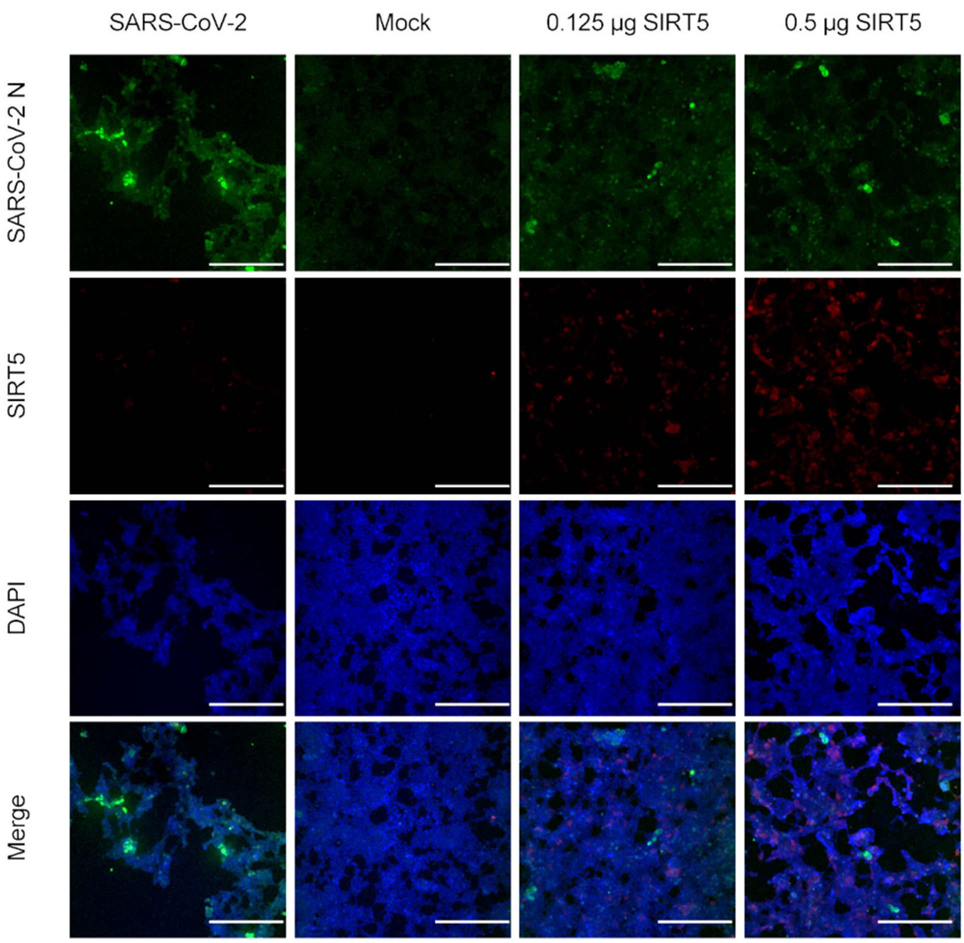

Fig. S13. SIRT5 reduced SARS-CoV-2 proliferation in HEK293T-hACE2 cells by IFA. After transfection of vector HA-SIRT5, viral N protein (green) and SIRT5 (red) of HEK293T-hACE2 cells were detected, nucleic DNA was stained by DAPI. Scale bar, 100 μm.

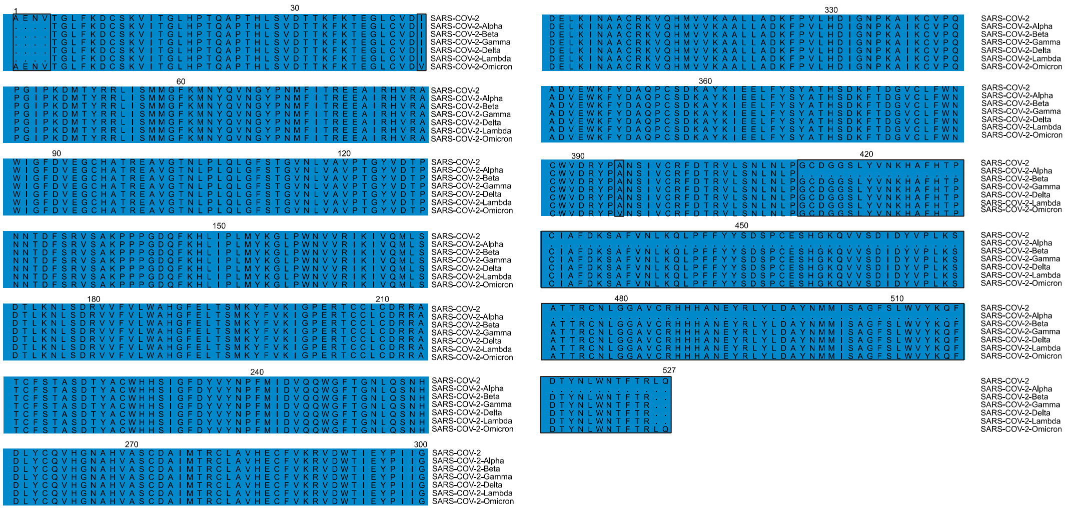

Fig. S14. Sequence coverage in protein analysis. NSP14 proteins of SARS-CoV-2 and other coronaviruses are shown in the sequence alignment.

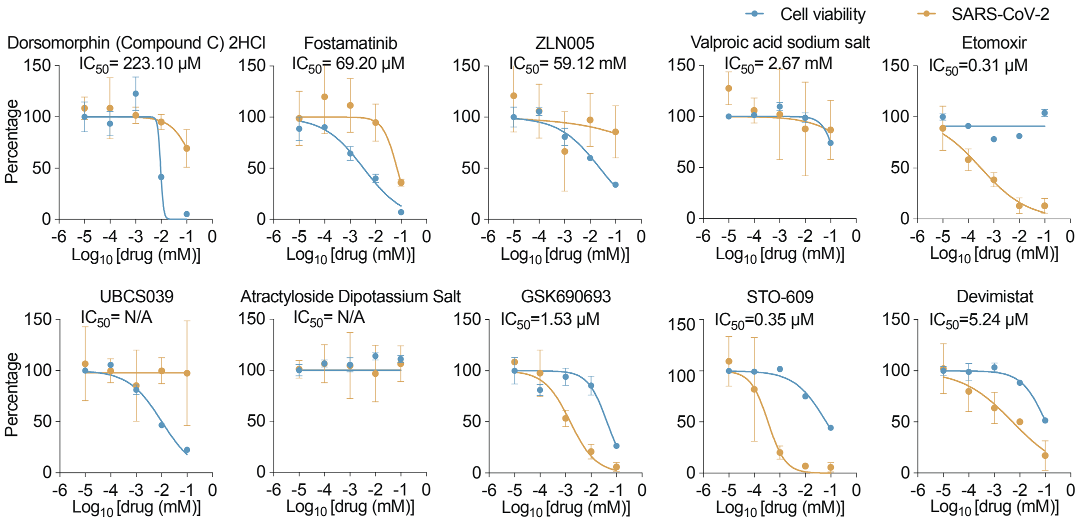

Fig. S15. Cell viability and determination of IC_{50} in Caco-2 cells. Cells were infected with SARS-CoV-2 (MOI = 0.01) for 48 h, and IC_{50} were detected by RT-qPCR. The cell viability was measured by cell counting (CCK-8) assay without viral infection. The orange line indicates the effects in infection measured at each drug dose. The blue line shows cell viability at each drug dose. Each drug had three independent repeated experiments. Error bars represent the mean ± SEM of three biological replicates.

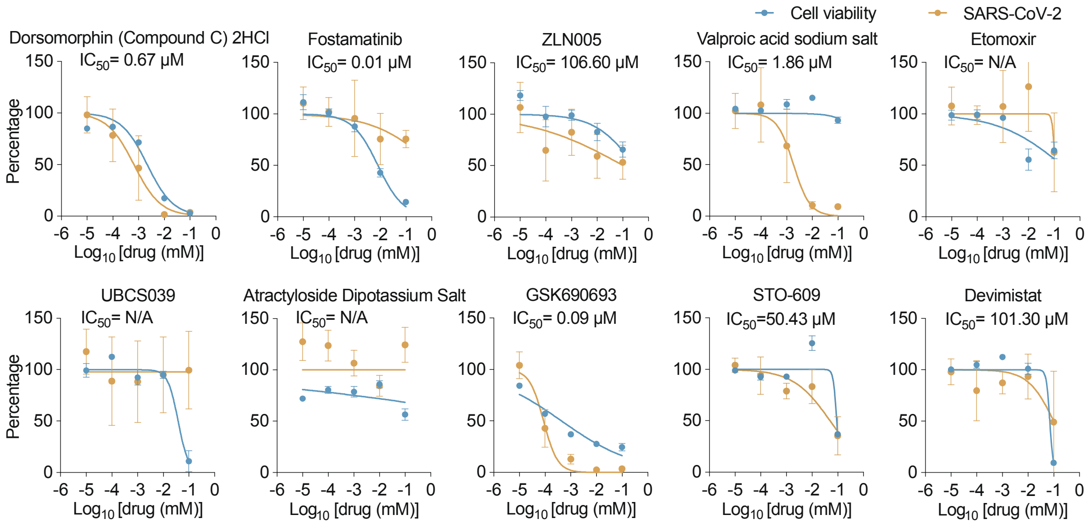

Fig. S16. Cell viability and determination of IC_{50} upon viral infection in HEK293T-hACE2. Cells were infected with SARS-CoV-2 (MOI = 0.01) for 48 h, and the cell viability and IC_{50} were detected by a cell counting (CCK-8) assay and RT-qPCR. The orange line indicates the effects in infection measured at each drug dose. The blue line shows cell viability at each drug dose. Each drug had three independent repeated experiments. Error bars represent the mean ± SEM of three biological replicates.

A

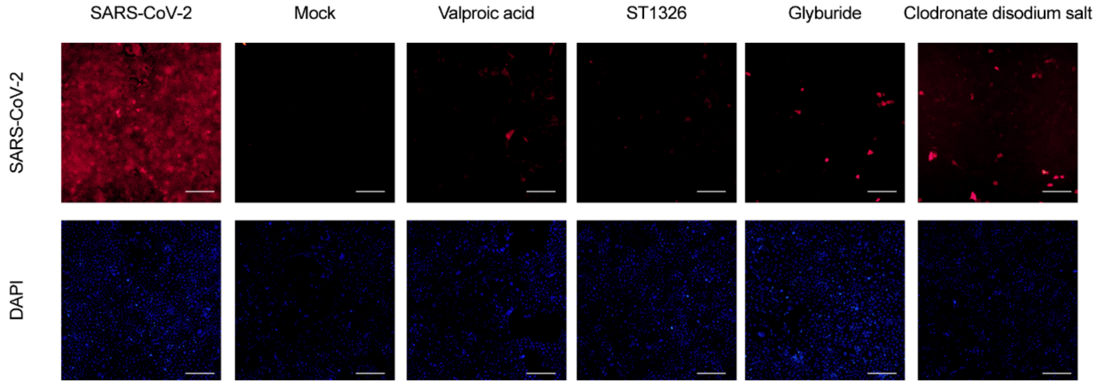

B

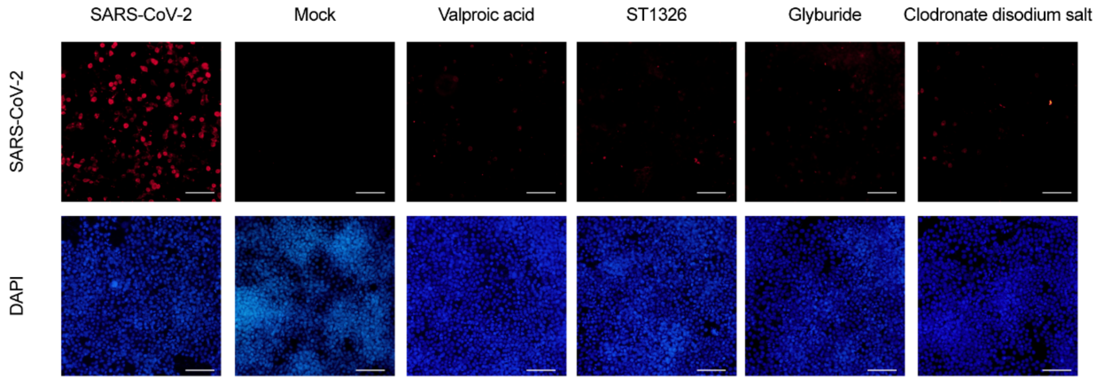

Fig. S17. Inhibitors of protein succinylation in host cells reduce virus replication detected by IFA. (A-B) After Caco-2 (A) and HEK293T-hACE2 (B) cells were infected with SARS-CoV-2 (MOI = 0.01) for 48 h, virus was detected by IFA. Cells were stained for N protein (red) to show SARS-CoV-2 levels and the nuclei were stained with DAPI (blue). Each drug had three independent repeated experiments (representative of two biological independent samples). Scale bars, 200 μm.

Table S1. Antiviral effects of the selected drugs or compounds in this study

Drug	Function	CC_{50} (μM, Caco-2)	IC_{50} (μM, Caco-2)	SI (Caco-2)	CC_{50} (μM, HEK293T-ACE2)	IC_{50} (μM, HEK293T-ACE2)	SI (HEK293T-ACE2)	FDA information	Clinic trial
Dorsomorphin (*Compound C*) *2HCl*	SIRT5 activator	9.563	223.1	0.0429	2.175	0.6653	3.2692	N/A	N/A
GSK690693	SIRT5 activator	42.3	1.533	27.5930	0.4369	0.0872	5.0086	N/A	N/A
STO-609	SIRT5 activator	66.64	0.3457	192.7683	92.08	50.43	1.8259	N/A	N/A
Fostamatinib	SIRT5 activator	3.361	69.2	0.0486	8.123	1131	0.0072	FDA approved	Phase 3
Valproic acid (*VPA*)	SIRT5 activator	335.2	0.3474	964.8820	>100	3.035	N/A	FDA approved	
ZLN005	SIRT5 activator	24.27	59120	0.000410 521	293.1	106.6	2.749531	N/A	N/A
Valproic acid sodium salt	SIRT5 activator	190.8	2666	0.071567 892	1930	1.863	1035.9635	N/A	N/A
UBCS039	SIRT6 activator	10.22	N/A	N/A	38.16	N/A	N/A	N/A	N/A
Etomoxir sodium salt	CPT1A inhibitor	150.5	0.8233	182.8009	274.6	2.1	130.7619	N/A	N/A
Etomoxir	CPT1A inhibitor	>100	0.3124	>320.102 4	206.1	N/A	N/A	N/A	N/A
ST1326	CPT1A inhibitor	36.5	0.86	42.4419	14.23	0.3014	47.2130	N/A	N/A
Glyburide	CPT1A inhibitor	794.1	0.8526	931.3863	32.77	0.6214	52.7358	FDA approved	
Atractyloside Dipotassium Salt	SLC25A5 inhibitor	>100	N/A	N/A	>100	N/A	N/A	N/A	N/A
Clodronic acid disodium salt	SLC25A5 inhibitor	>100	0.771	>129.701 6	231.5	0.317	730.2839	N/A	N/A
Empirical Formula (*Hill Notation*)	SLC25A5 inhibitor	2035	6.818	298.4746	2024	1.336	1514.9701	N/A	N/A
Devimistat (*CPI-613*)	KGDHC inhibitor	105.7	5.235	20.1910	>100	101.3	>0.9871	N/A	Phase 3

*Results are shown as mean ± SD of three biological replicates.

Supplemental Datasets

Dataset S1. Protein MS intensity of different proteins during virus infection.

Dataset S2. Protein MS intensity of different succinyl-proteins during virus infection.

Dataset S3. Read count of the different transcripts during virus infection.

Dataset S4. Fold change of total proteins for cluster analysis.

Dataset S5. Fold change of succinyl-proteins for cluster analysis.

Dataset S6. KEGG enrichment for differentially expressed succinylated proteins.

Dataset S7. Fold change of the succinylated site levels for cluster analysis.

Dataset S8. Antiviral effects of the selected drugs or compounds in this study.

SI References

1. T. Wu *et al.*, clusterProfiler 4.0: A universal enrichment tool for interpreting omics data. *Innovation (N Y)* **2**, 100141 (2021).
2. A. Stukalov *et al.*, Multilevel proteomics reveals host perturbations by SARS-CoV-2 and SARS-CoV. *Nature* **594**, 246-252 (2021).
3. P. J. Mullen *et al.*, SARS-CoV-2 infection rewires host cell metabolism and is potentially susceptible to mTORC1 inhibition. *Nat Commun* **12**, 1876 (2021).
4. K. S. Greene *et al.*, SIRT5 stabilizes mitochondrial glutaminase and supports breast cancer tumorigenesis. *Proc Natl Acad Sci U S A* 10.1073/pnas.1911954116 (2019).
